# Identification of microplastic fibres released from COVID-19 test swabs with Raman imaging

**DOI:** 10.1186/s12302-023-00737-0

**Published:** 2023-05-06

**Authors:** Cheng Fang, Yunlong Luo, Clarence Chuah, Ravi Naidu

**Affiliations:** 1 Global Centre for Environmental Remediation (GCER), University of Newcastle, Callaghan NSW 2308, Newcastle, Australia; 2 Cooperative Research Centre for Contamination Assessment and Remediation of the Environment (CRC CARE), University of Newcastle, Callaghan NSW 2308, Newcastle, Australia; 3 Flinders Institute for NanoScale Science and Technology, College of Science and Engineering, Flinders University, Adelaide, South Australia 5042 Australia

**Keywords:** COVID-19 testing kit, Swab, Microplastic, Titanium oxide particle, Algorithm, Raman imaging

## Abstract

**Background:**

COVID-19 pandemic is not yet over, and it has been generating lots of plastic wastes that become a big concern. To catch the virus, for example, no matter via antigen or PCR test, swab is generally used for sampling. Unfortunately, the swab tip is commonly made of plastics, and thus it can be a potential source of microplastics. This study aims to propose and optimise several Raman imaging to identify the microplastic fibres released from different COVID-19 test swabs.

**Results:**

The results show that Raman imaging can effectively identify and visualise the microplastic fibres released from the swabs. In the meantime, on the surface of the fibres, additives such as titanium oxide particles are also captured for some brands of swabs. To increase the result certainty, scanning electron microscope (SEM) is first employed to get the morphology of the released microplastic fibres, along with Energy-dispersive X-ray spectroscopy (EDS) to confirm the presence of titanium element. Then, Raman imaging is advanced to identify and visualise the microplastics and titanium oxide particles, from different characteristic peaks in the scanning spectrum matrix. To further increase the imaging certainty, these images can be merged and cross-checked using algorithms, or the raw data from the scanning spectrum matrix can be analysed and decoded via chemometrics, such as principal component analysis (PCA). Beyond the advantages, the disadvantages of the confocal Raman imaging (affected by focal height) and algorithms (non-supervised calculation) are also discussed and intentionally corrected. In brief, the imaging analysis (particularly the combined SEM with Raman) is recommended to avoid the possible result bias that might be generated from the single spectrum analysis at a selective but random position.

**Conclusions:**

Overall, the results indicate that Raman imaging can be a useful tool to detect microplastics. The results also send us a strong warning that, if we worry about the potential microplastics contamination, we should be cautious to select the suitable COVID-19 testing kits.

**Supplementary Information:**

The online version contains supplementary material available at 10.1186/s12302-023-00737-0.

## Introduction

Since the onset of the COVID-19 pandemic, healthcare manufacturers have been ramping up production to meet the surging demand on disposable medical supplies, such as personal protective equipment (PPE), testing kits and consumables [1]. While these items play the essential roles in mitigating and slowing down the spread of the virus, some potential negative impacts have been highlighted already [2]. As a large portion of the disposable medical supplies are made of plastics, inappropriate disposal and mismanagement of the pandemic waste aggravate the existing plastic pollution crisis and create an extra burden for ecosystems and environment [3]. Furthermore, these single-use plastics are possible sources of microplastics (fragments < 5 mm) and nanoplastics (< 1 µm), which can be potentially hazardous to a wide range of living organisms including humans [4, 5]. Therefore, it is important to understand the possibility of microplastic formation when using the plastic-based medical products.

To catch the COVID-19 virus via polymerase chain reaction (PCR) or rapid antigen test (RAT), testing kits have been widely employed. In most of the kits, swab is used to collect samples from people’s nostrils and throats. A swab generally constitutes two parts, a handle (applicator shaft) and a tip with adsorbing materials. The adsorbing materials are commonly made of synthetic fibres, such as nylon and polyester, or cotton [6, 7]. To sample the COVID-19 virus, a swab is inserted into the left and right nostril or mouth, and rotated several times against nasal or throat wall [8]. This sampling process creates contact or friction between the swab and the tissue, which may shed and release microplastics in the form of fibre fragments from the tip. The released microplastic fibres can later enter the human body and pose a health risk in potential. Unfortunately, no research has been conducted to investigate this issue.

To evaluate the release of microplastics from swabs, a reliable analytical method is required. Raman spectroscopy is a promising vibrational spectroscopic technique that can characterise various materials via the “fingerprint” Raman spectrum. The advantages of Raman spectroscopy, such as minimal sample preparation, non-destructive/label-free analysis and insensitivity to water, have been well-demonstrated [9]. Recent advances in Raman spectroscopy facilitates its application in numerous fields of research including microplastics, such as via Raman imaging [10].

For Raman imaging, a Raman spectrometer is integrated into a regular optical microscope, which results in a high magnification of the target and the subsequent Raman spectroscopic mapping. Compared with Fourier transform infrared spectroscopy (FTIR)-based imaging, Raman imaging has a considerably smaller diffraction-limited spatial resolution (less than 1 µm *vs.* more than 20 µm) to analyse the smaller targets, due to the shorter wavelength of the excitation light [11, 12]. Furthermore, using confocal Raman imaging, the diffraction limit of Raman’s excitation laser can be potentially broken through, enabling the analysis of nanoplastics down to 100 nm [13].

Raman imaging commonly acquires tens to thousands of Raman spectra as an array by laser-scanning an area of interest. Once the spectra are collected, they are analysed to map Raman images that offer not only chemical characterisation but also spatial and morphological information (e.g., size and shape) [14]. More importantly, the scanning on the target surface can effectively avoid result bias from the single spectrum that only reflects the situation at the spectrum collection position. That is, the single spectrum (point analysis) is generally collected from a random position on the target surface under a pre-condition that the chemical component in the target is uniformly distributed. Unfortunately, this pre-condition might be not true for microplastics analysis, no matter for the plastics component or for the co-existent ingredients. Imaging (area analysis) can scan the target to avoid this bias result, by uniformly collecting tens to thousands of spectra from an area.

During the scanning process, the spectra are collected as an array or a spectrum matrix. To convert the matrix’s raw data into images, several methods can be used. The most straightforward one is to map the signal intensity at a wavenumber or frequency (usually a characteristic peak of the suspected target) [15]. To improve the imaging certainty, images created from several characteristic peaks can be merged and cross-checked using algorithms [16]. Raman images can also be produced via chemometric tools, such as principal component analysis (PCA) [17]. PCA has the ability to identify the major spectral variations of the scanned target, to produce image via the whole set of spectrum rather than from just a few selected characteristic peaks [18]. A further correlative analysis can be performed to automatically and digitally link these spectral variations to certain targets [19]. Despite the recent advances in Raman imaging, more validation studies are required to evaluate its robustness in the microplastic analysis.

Given the urgency of the microplastic contamination issue, this study is implemented with dual objectives: to validate and refine different Raman imaging methods, and to explore and quantify the microplastics and nanoparticles released from an unreported source in relation to the COVID-19 testing kits. Specifically, we adjust logic-, algebra- and PCA-based algorithms to aid the imaging of microplastic fibres as well as the additives shed from swabs during the COVID-19 sampling process. We further adopt a super-high-resolution (magnification) method to analyse the nano-sized target in detail that might be ignored at a low magnification, particularly for single spectrum analysis (point analysis *vs.* area analysis) that can lead to the bias result. We check several swabs in the COVID-19 testing kits for comparison. Overall, the findings from this study will not only contribute to the Raman-based microplastic research, but also help to understand risks associated with swab sampling for diagnosis of diseases including but not limited to the COVID-19.

## Materials and methods

### Chemicals and samples

All chemicals including ethanol and acetone were purchased from Sigma-Aldrich (Australia) and used as received. Milli Q (MQ) water (> 18 MΩ cm) was used for the analysis. The COVID-19 testing kits were either provided by South Australian government, Xiaman government (China), or purchased from local supermarkets (Woolworths, ALDI and Pharmacy, Australia) and eBay (Australia), and shown in Additional file 1: Figure S1. There are many brands of self-testing kits in Australia, such as ~ 60 approved by TGA (Therapeutic Goods Administration, Australia). Among them 45 are made in China, rests in Korea (3), Singapore (2), USA (2), UK (2) and other countries. What we have checked contains one made in Australia (#1) while the rests made in China (#2–7), including Innoscreen (#1), CLUNGENE^®^ (#2), Hough (#3), RightSign^®^ (#4), ALLTEST™ (#5); Flowflex™ (#6) and LABCARE (#7, from China). The last one works as a throat swab for PCR test while the rests as nasal swabs for RAT test. Sample #1 was focused on this study and we then expanded the check to the rests.

All virgin microplastics (beads or pellets, usually with diameters < 1 mm) including polystyrene (PS), polyethylene terephthalate (PET), polyethylene (PE), polyvinyl chloride (PVC) and polypropylene (PP) were purchased from Sigma-Aldrich (Australia) and used as received. Several Raman spectra were extracted from the database when the standard plastic targets were not available, inducing polyamide (PA or PA 6), poly(methyl methacrylate) (PMMA), polycarbonate (PC, be careful, it is different from the principal components of PCA, marked as PC# in this study, such as PC1, PC2 etc.), polyurethane (PUR), acrylonitrile butadiene styrene (ABS), rubber, poly(tetrafluoroethylene) (PTFE) and cotton.

Before check, glass slides or aluminium foil were cleaned with ethanol, acetone and MQ water. When the surface was still wetted with a thin layer of MQ water, a COVID-19 testing kit was opened on-site and the nasal swab was taken out to touch the not-yet-dried surface gently, as suggested by the kit introduction, to mimic the sampling process. After 2–15 s mimicked sampling, some fibres can be observed on the glass or aluminium surface. If not, the swab tip can be cut into small pieces for check using a stainless scissors that have been previously cleaned. Once dried, it was ready for Raman check.

The wetted glass surface is different from nasal or throat, such as the absence of sticking saliva. To increase the sticking interaction, we employed a carbon tape. In this case, the carbon tape was prepared on a scanning electron microscope (SEM) holder. The covering paper was peeled off on-site and a swab was employed again to gently touch the carbon tape surface, to mimic the sampling process. Although the mimicked process is different from the real situations, this study can lead to a better understanding on the likelihood of the releasing microplastics from the COVID-19 testing kits and the similar products.

### Checking protocols

An SEM (Zeiss Sigma VP) was used to characterise the morphology of the released microplastics and particles, in addition to energy-dispersive X-ray spectroscopy (EDS) detection. The sample was sputter-coated with a thin layer of platinum (~ 6 nm) to increase the conductivity. The accelerate voltage was 10–20 kV with a working distance of 5–10 mm [20].

The Raman study was following the previous report [21, 22]. In brief, Raman spectra were recorded in air using two confocal Raman microscopes (Alpha 300RS/WITec, Germany; and DXRxi/ThermoFisher, USA) equipped with 532 nm laser diode (< 30 mW). A charge-coupled device (CCD) detector was cooled at − 60 °C to collect Stokes Raman signals under an objective lens (100 × , or others, such as 40 ×) at the room temperature (~ 24 °C).

To map the Raman image, the laser was scanning the target surface to collect the spectrum at each pixel or point, as a scanning spectrum matrix. DXRxi enables the terrain mapping and imaging by collecting the terrain map in advance, which will be detailed later. The spectrum matrix was analysed using WITec Project or DXRxi OMNICxi imaging software, to pick up the net intensity of the characteristic peak for image mapping, or via the correlation with a reference spectrum (DXRxi). The former is preferred. At the selected peaks, the interference which might originate from the background noise (such as fluorescence), or organic matter, can be intentionally avoided by subtracting the baseline of the collected Raman spectra to get the net intensity (the peak area or sum, after automatic integration via software). Alternatively, the scanning spectrum matrix can be analysed using the following algorithms.

### Imaging algorithms

#### Logic-based algorithm

From the Raman spectra matrix, several images can be simultaneously mapped from different peaks in the same spectrum. Two or more images, which correspond to two or more different characteristic peaks, can be merged, such as by logic-OR, or colour-channel-merge, using ImageJ software. In the latter case, the original colour can be maintained to distinguish their individual contributions.

In the case of “logic-OR”, any mapped signal (or dot, pixel) from any image (parent images) will be picked up and merged into a new image (daughter image). Obviously, any “bias, false” and noise from any parent images might be picked up and appear in the daughter image too. On the contrary, in the case of “logic-AND”, only the simultaneously mapped signal in all parent images can be picked up and merged into a daughter image.

#### Algebra-based algorithm

Two or more images, no matter mapping the Raman intensity or the PC loading coefficients (to be discussed below), can be merged using algebra functions with an increased flexibility, using Origin software [23]. Before merge, we normalise the colour value to 0–255 or the loading coefficient to 0–1, to avoid bias. We then merged them using various algebra functions.

For example, the Raman peak heights can act as the weighting factors towards merge [24]. Taking PA as an example, we can set the weighting factors to be 0.07, 0.13, 0.14, 0.24, 0.19 for the images mapped at ~ 1050 cm^−1^, ~ 1130 cm^−1^, ~ 1300 cm^−1^, ~ 1450 cm^−1^ and ~ 1630 cm^−1^, respectively. These weighting factors are the relative heights of PA’s Raman characteristic peaks, if we take the highest one at ~ 2910 cm^−1^ as 1. After merging, we normalise it back to 0–255 again for imaging. That is (0.07 × normalised image mapped at ~ 1050 + 0.13 × normalised image mapped at ~ 1130 + 0.14 × normalised image mapped at ~ 1300 + 0.24 × normalised image mapped at ~ 1450 + 0.19 × normalised image mapped at ~ 1630 + 1 × normalised image mapped at ~ 2910)/1.77. Similarly, we can simply average these characteristic peaks images, using (normalised image mapped at ~ 1050 + normalised image mapped at ~ 1130 + normalised image mapped at ~ 1300 + normalised image mapped at ~ 1450 + normalised image mapped at ~ 1630 + normalised image mapped at ~ 2910)/6.

#### PCA-based algorithm

The raw data from Raman scanning spectrum matrices were analysed by PCA in Origin (Pro 2022) software, as reported before [17, 21]. DXRxi data need to be saved as “h5” format first and then re-arranged in Origin by transposing and column-splitting. For Witec, each set of Raman spectrum was recorded in the wavenumber range of -200 cm^−1^ − 3700 cm^−1^ with 1024 readings (intensity data) at the corresponding wavenumbers as a column. For DXRxi, each set of Raman spectrum was recorded in the wavenumber range of 50 cm^−1^ − 3400 cm^−1^ with 1738 readings (intensity data) at the corresponding wavenumbers as a column. Therefore, the data array will be 1024 (rows) × scanning array (columns), or 1738 (rows) × scanning array (columns). The scanning array is controlled by the scanning area and the pixel size, such as 30 × 30, and is adjustable.

After deleting the data in the wavenumber range of < 200 cm^−1^ (from Witec) to remove the residue laser, the rest Raman signal data were involved in the PCA analysis, using parameters including “correlation analysis”, “exclude missing values of listwise”, and “quantities of compute including eigenvalues and eigenvectors”. The wavenumbers (or wavelengths) act as the “observation label” and are linked with the row sequence number. The principal components (PC#) were adjusted according to the estimated number of suspected targets could be located in the area scanned. Normally, up to five principal components were extracted.

After PCA, the PC scores (as *y-*axis) at their individual eigenvalue percentage of variance were combined with the Raman wavenumber (as *x*-axis, via the sequence number of the row) to regenerate a curve, called a PCA spectrum, to mimic the Raman spectrum. This mimic curve is justified to identify the plastics and other targets, by comparing it with the standard Raman spectrum of the suspected item, if available. Each loading coefficient of the PCs (as *z*-axis) is combined with the mapping pixel’s position (as *x–y*-axis, via the sequence number of the column) to map an image, called a PCA image. Depending on the presentation orientation of the raw data array, the scores and the loading coefficients can be swapped or transposed.

PCA can also identify the target via correlation by combining its spectrum with the standard spectra, akin to indexing [23, 25]. To avoid the comparison bias and to increase the accuracy, we pre-treat all the Raman spectra, using techniques including cosmic ray removal, smoothing, baseline correction, interpolating to adjust the wavenumber (*x*-axis) and normalising the intensity (*y*-axis). After the pre-treatment, we then run an PCA analysis on this spectrum matrix. The generated correlation matrix enables the assignment of suspected target, automatically and digitally.

## Results and discussion

### SEM and EDS

**Fig. 1:**
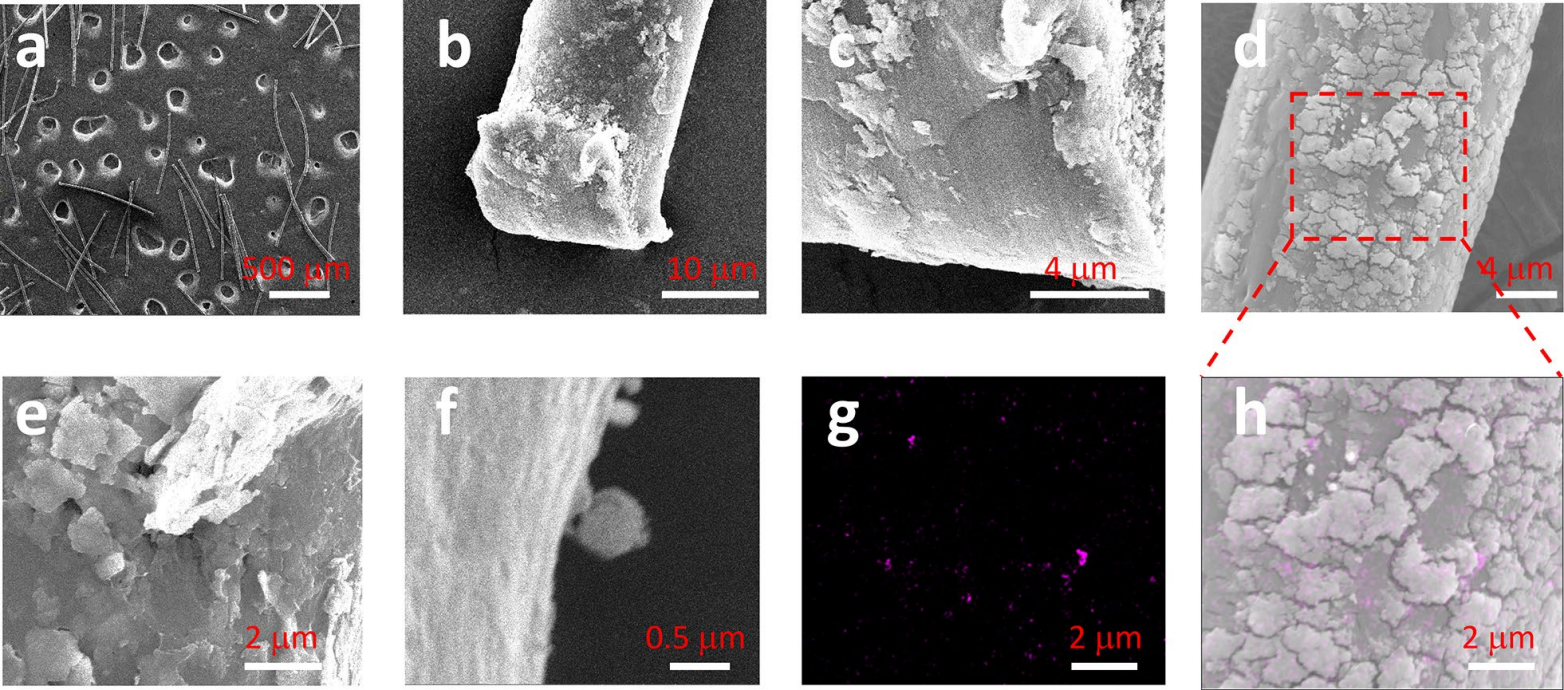
SEM images recorded on carbon tape surface for Sample #1’s swab tip. (**a**) is an overview, (**b**, **c**) detail the fibre end and (**d**–**f**) show the middle part. (**g**) maps element titanium and (**h**) overlaps (**g**) onto the SEM image

Figure 1 shows the SEM images of the released items from a swab tip. The swab’ tip is a brush, as shown in Additional file 1: Fig. S1, containing many fibres. The fibres can be easily released, as shown here.

We also note there are particles on the fibre surface, no matter at the end of fibre (Fig. 1b, c) or in the middle part of fibre (d–f). (c) suggests that there might be a coating layer on the fibre surface, which might be functioned for the COVID-19 sampling [26, 27]. That is, the layer containing particles is either formulated to realise the sampling or medical function or added as additive in the fabrication process. Further testing is required to determine the material of this coating layer, which is demonstrated in the following section [28]. The particles or debris are observed in the size range of 0.2–2 μm and can be peeled off, as suggested in (c, e, f).

To identify the particle, EDS has been collected and the results are presented in Additional file 1: Fig. S2. At some positions (not every position), where we have collected, EDS suggests the presence of titanium. Once titanium is mapped, an image in Fig. 1g is generated. It can overlap onto the SEM image to get an image (h). The non-uniform distribution of titanium via particles is observed. That is, the single position analysis of an EDS might bypass the titanium, resulting in bias. In this case, imaging analysis should be conducted (area analysis *vs.* point analysis).

### Raman identification: PA plastic

**Fig. 2:**
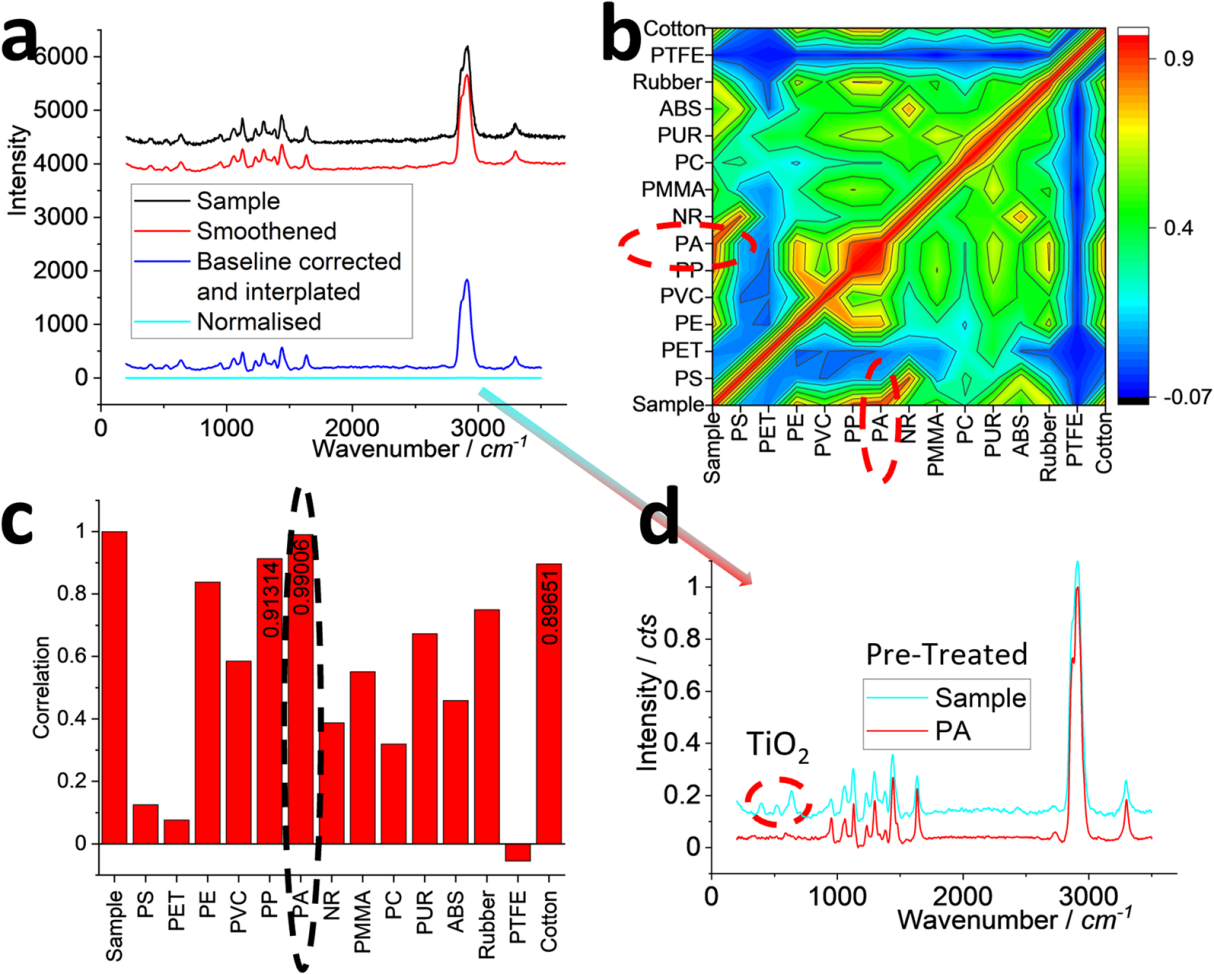
Raman spectra (**a**, **d**), correlation matrix (**b)** and correlation values (**c**) of a typical spectrum collected from the released fibres. In (**a**), the collected sample’s spectrum is pre-treated towards identification, after intensity off-setting for presentation. Similarly, all the spectra including the sample and 14 items (13 common plastics and cotton) were subjected to the pre-treatment and then participate PCA to generate a correlation matrix in (**b**). The correlation value of the sample with 14 items is listed in (**c**). The direct comparison between the spectra of sample (after pre-treatment) and the standard spectrum of the assigned PA is presented in (**d**), after intensity off-setting for presentation. The assignment is indicated by circulating in (b, c)

To identify the materials of the swab fibres, we take a typical Raman spectrum for analysis, as shown in Fig. 2a. To accurately identify it, we pre-teat the spectrum, including curve smoothening to remove the random noise, baseline correction to off-set the possible fluorescence background, wavenumber interpolation to standardise the *x*-axis, intensity normalisation (to 0–1) to standardise *y*-axis [23].

Similarly, we also pre-treat all the spectra in our data base, including 13 common plastics and cotton. We then run PCA to generate a correlation matrix, as shown in Fig. 2b. The “sword-like” pattern is symmetric along the diagonal, where each item is correlated with itself to yield a correlation value of 1. Only the left column or the bottom row is meaningful for the sample [21]. We can see that it has the highest correction with PA or nylon.

The correlation values can be extracted and presented in Fig. 2c. The highest one is 0.99006 for PA, with the second one for PP at 0.91314, and the third one for cotton at 0.89651. Taking the highest one, we can assign it to PA, automatically and digitally. The direct comparison between the sample and PA is provided in (d). A well-match can be observed, to support the above assignment. There are several circulated peaks that are not from PA, which leads to the correlation value slightly differing from 1. These peaks come from additives or co-ingredients from the nanoparticles observed in Fig. 1 [29], such as titanium oxide (TiO_2_), and will be discussed later.

Similarly, we also identify the swab bar (handle) and the testing cartridge as natural rubber (NR), as suggested in Additional file 1: Figs. S3, S4. However, the swab tip is focused herein, because it can release fibres that can be categorised as microplastics.

### Raman imaging

#### Visualisation of PA fibres

**Fig. 3:**
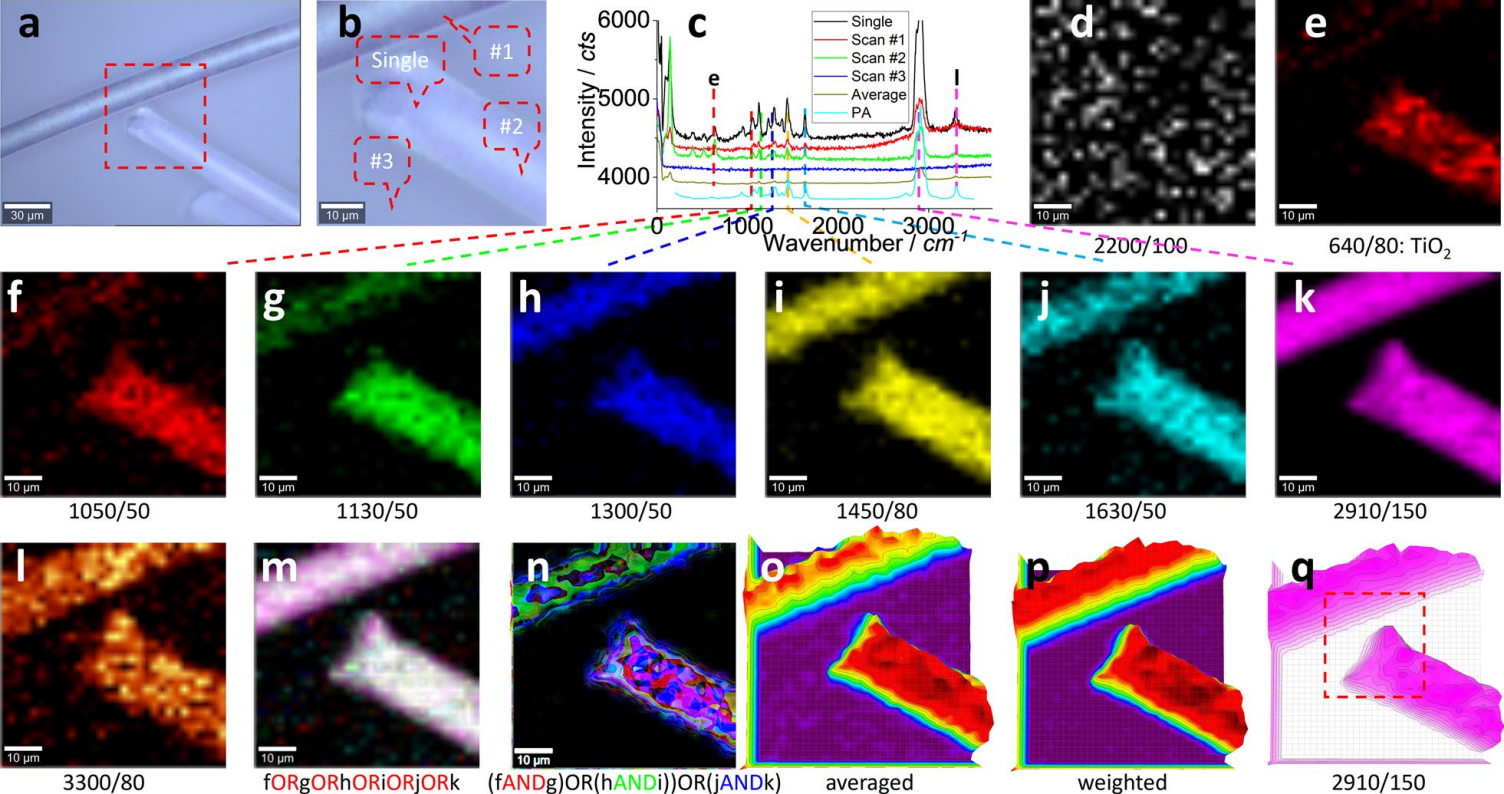
Photo images (**a**, **b**), typical Raman spectra (**c**) and images (**d**–**q**). The squared area in (**a**) of 60 μm × 60 μm was zoomed in as (**b**) and scanned. Raman spectra were collected under an objective lens of 40 × , integration time of 1 s for each pixel of 2 μm × 2 μm (to create a matrix of 30 × 30). (**c**) shows the Raman spectra of PA, to compare with 1 single spectrum (10 s integration) and 3 typical scanning spectra (1 s) collected from the marked positions in (**b**), and the average spectrum of 900 (30 × 30) spectra. The intensity images (**d**–**l**) are mapped at a blank wavenumber window (**d**), a characteristic peak ofTiO_2_ (**e**) and seven characteristic peaks of PA (**f**–**l**), as marked under each image (and the peak width), after 10% colour off-setting. (**m**, **n**) and (**o**, **p**) merge images (**f**–**k**) using either logic-based algorithm (m, n) or algebra-based algorithm (**o**, **p**), as suggested under the images. (**q**) is another version of (**k**), using a 3D presentation and white a background. The squared area is further checked in Fig. 4

The above assignment is based on a single spectrum, the point analysis can lead to bias in potential. That is, the assignment is only valid at the spectrum collecting point, other area beyond this point is unknown. Imaging analysis has been recently developed, thanks to the advancements on the technologies and the computation capacity, to visualise the target from chemical point of view. That is, via scanning the target surface to collect the spectrum at the scanning area/array as a scanning spectrum matrix (a hyperspectral matrix), the spectrum information can be mapped at the scanning position to generate an image. The assignment certainty or the analysis sensitivity can be significantly increased, from a statistical point of view. The results are shown in Fig. 3.

Figure 3a, b shows the photo images for scanning. During the scanning process, the collected typical spectra are presented in (c), from the positions marked in (b). The single spectrum taking a longer integration time (10 s *vs.* 1 s) (position marked too) is also presented, along with the standard spectrum of PA. Basically, the single spectrum and Scan #1 spectrum can be assigned to PA again. Spectrum of Scan #2 can also be assigned to PA, although the intensities of PA peaks are weak and there are several strong peaks in the low wavenumber range that are not from PA. Scan #3 was collected from the blank area as the spectrum background. The average spectrum (900, or 30 × 30) is also shown. The random noise is significantly decreased, and the characteristic peaks of PA are also visible, suggesting the presence of PA in this scanning area and the increased signal–noise ratio of imaging analysis.

Before we map PA via the characteristic peaks, we first map a blank wavenumber window or channel, where PA has no signal. The image is shown in Fig. 3d and can act as an internal reference image to visualise the image background. In (e), a peak that does not originate from PA is mapped, but likely from TiO_2_ [29], which will be further discussed below, along with the weakly patterned upper fibre.

The characteristic peaks of PA are mapped as images in Fig. 3f–l. All of them generate clear patterns that look similar with (b), suggesting the fibres in (b) are PA, rather than just at the point to collect the single spectrum that has been analysed in Fig. 2. That is the main advantage of Raman imaging.

The images in Fig. 3f–k can be merged together to further increase the assignment certainty. To this end, we employ a logic-based algorithm. The simple way is to merge them via colour channels, including, red (f), green (g), blue (h), yellow (i), cyan (j) and magenta (k), using software ImageJ. (l) is not selected, because six images might be enough for this kind of merge, to cross-check the presence of PA. The resulted image is shown in (m), where the white pattern contains all the contributions from the six peaks (channels) separately, implying an increased assignment certainty, or an increased signal–noise ratio.

This merge works like logic-OR, but the original contributions from different image (channel) can be maintained and visualised. Another merge way is to mix the calculation, including logic-OR and logic-AND. The benefit is, while OR can pick up any signal appeared in any “parent” image (signal enhanced but noise survived), AND can only pick signal just simultaneously appeared in all parent images (signal survived but noise decreased). The mixed calculation can thus inherit both advantages, signal enhanced and noise decreased. The resulted image is shown in Fig. 3n. It looks similar with (m), but the certainty has been increased to assign it to PA.

The logic-based algorithm calculates the contribution from the parent images via colour threshold. In Fig. 3f–k, the peak intensity for mapping is different for each characteristic peak, as presented in (c). The different intrinsic peak intensity means the image has different colour threshold, which might be the reason why the colour is not uniform in merged pattern in images (m, n). To overcome this shortage, an algebra-based calculation can be more flexible to merge them. The results are presented in (o, p).

The simple algebra math is to average them, as presented in Fig. 3o. Obviously, the different intrinsic peaks’ contributions have not been differentiated by the average. Because the different peak has different intensity, we can take the intensity (relative peak height) as the weighting factor to merge them in (p). It looks similar with (o), but the upper fibre is more clearly presented, suggesting the improvement.

The above algorithm-merge can increase the assignment certainty. Actually, the signal–noise ratios for all characteristic peaks are good in Fig. 3, particularly for image (k), another version of which is presented as (q), where the strongest peak is mapped and dominates the merge. Once assigned via images in Fig. 3m–p, we can take the strongest peak to visualise the PA via (q), in order not to lose the signal, which is employed below.

#### Focusing height effect

**Fig. 4:**
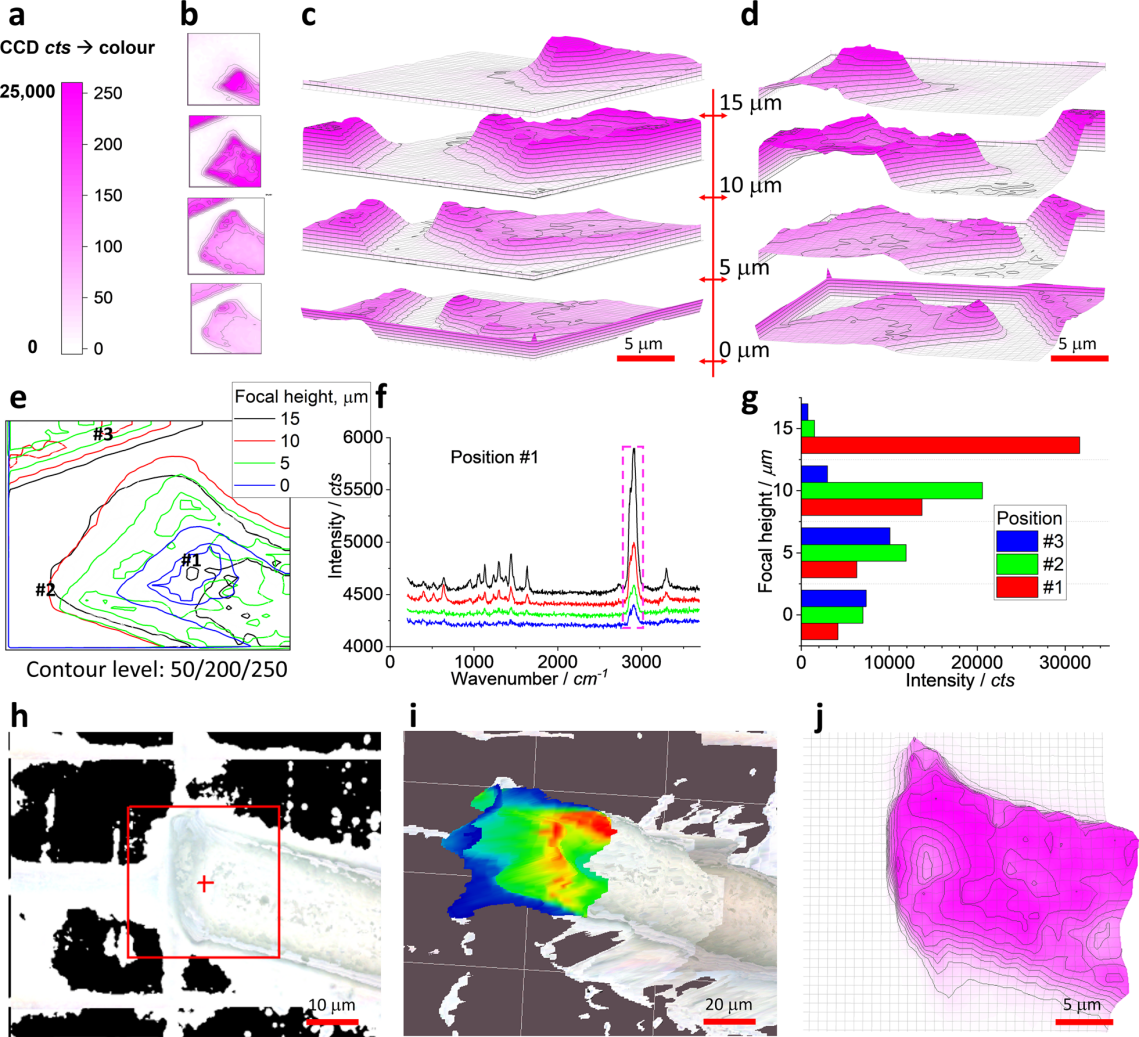
Effect of focusing height. The scanning area of 30 μm × 30 μm is squared in Fig. 3q and Raman spectra are collected under an objective lens of 100 × , integration time of 1 s for each pixel of 1 μm × 1 μm (to create a matrix of 30 × 30). (**a**) is the colour scale bar. (**b**) maps the PA via peak of ~ 2910 cm^−1^, at different focusing heights along *z*-axis. (**c**, **d**) present them together at the indicated physical heights, using 3D presentation and white background, from different angle of view. (**e**) merges them together in the scanning plane (*x-/y*-axis) using their contours. At the marked position (#1), the collected spectra are shown in (**f**). (**g**) compares the peak intensities at ~ 2910 cm^−1^ for the marked positions #1–3, once the focusing heights are changed. (**h**) shows the terrain map for imaging. (**i**) is the mapped Raman image overlapped on the terrain map, using the rainbow colour. For comparison, (**j**) shows an image mapped without the terrain map

Confocal Raman can effectively collect the signal from the focal plane. Off-focal plane has a declined contribution. The released fibre has a 3D structure like a lay-down cylinder, which might lead to the false Raman image under the confocal setup. To check this assumption, we fix the scanning *x-/y*-plane squared in Fig. 3q, but change the focusing height along *z*-axis and repeat the scanning. The results are shown in Fig. 4.

Figure 4a shows the colour scale bar for map, to convert the signal intensity to colour. At four different focusing heights along *z*-axis, the images are presented in (b). We can see the patterns are different, due to the changed focal plane. The mapped pattern can be presented as 3D, but the *z*-axis is pseudo, because it is generated by the signal intensity, rather than the physical height. The physical height is changed at four different positions, as marked in the middle of (c, d).

Once put together, Fig. 4c, d are produced, from different angle of view. The patterns are different even in the same *x-/y*-plane. Note the bottom image has the boundary effect due to the image exporting format that has not been corrected, while the rests have been corrected. Therefore, the focal position (*z*-axis) should be carefully selected to maximum the signal for mapping. However, the reality is that the target is neither exactly localised in the same plane nor have the same height, which might lead to the false or bias image. This situation gets serious when the target is big at *z*-axis, such as > 5 μm, once the objective lens of 100 × is employed. That is, along with the lateral resolution in the *x-/y*-plane (~ 300 nm, if defined by full width at half maximum (FWHM) of laser spot, *0.51λ/NA* or *λ/2NA or 0.61λ/NA*, *λ* is the laser wavelength of 532 nm, while *NA* is the numerical aperture of the objective lens of 0.9 for 100 ×), the laser also has a spatial resolution along the *z*-axis (~ 1300 nm, if defined by *2λ/NA*^*2*^) [30]. The scanning in the fix plane or the same *x-/y*-axis thus can lead to bias image at different *z*-axis, as shown here.

The patterns mapped at different physical heights have the pseudo *z*-axis via Raman intensity, as mentioned. Ideally, at each height, the mapped image should be like a slide to see the cross section of the focal plane. The reality is, although the laser is focused on the physical plane, the up/down part at the off-focal plane is also illuminated by the off-focal laser. Consequently, the scattering signal is also likely collected to contribute the imaging. In the meantime, although the laser has a permeating depth [30, 31] in a hope to reach the focal plane (if focused on depth, not on the surface), it might be blocked by the upper part of the target and cannot reach the focal plane. In this case, the target surface is also illuminated towards the Raman scattering. This is the reason why we presented the images as 3D at each focal height, even some false or bias might be there.

Once all images are projected onto the same *x-/y*-plane, Fig. 4e is generated, via the contour lines at colour values of 50/200/250, at each focal height. Again, we can see the different pattern, which means the possible false image, or not the exact physical size of the target at least. On the left-top corner, once the focal position is left-up, the pattern of the upper fibre in Fig. 3 shrinks and disappears at height of 15 μm, as evidenced in (b). For the right-bottom fibre, the pattern changes too and depends on the focusing heights. As said, the fibre has a 3D structure of a cylinder, the confocal imaging might have difficulty to map the exact physical profile, meaning a false image in potential.

We can have a close look at the selected *x-/y*-position, to check the effect of the focal height. At the position #1, which is marked in Fig. 4e, the collected spectra are presented in (f). While the intensify has been off-set for clarification, we can see the net peak intensity (squared) increase with the increase of the focal height. Perhaps this position is the highest part (physically) in the scanning area squared in Fig. 3q. We can extract the intensity at peak ~ 2910 cm^−1^ and show as (g). Similarly, two more positions are analysed, and the data is also presented. Position #2 has the strongest intensity at ~ 10 μm, which might be its physical height. Position #3 has the strongest intensity at ~ 5 μm, which might be its physical height, relatively.

In brief, the confocal imaging can effectively collect the signal from the focal plane. However, the 3D structure of the target might not be mapped and imaged to reflect the exact physical profile. In this case, other approach should be selected, as shown in Fig. 4h, i.

To this end, we need the mapping parameters of the target surface, or a terrain map with *x-/y-/z*-axis. In Fig. 4h, we change the focal heights at every 2 μm to collect the photo images and generate the terrain map. Using this terrain map, the confocal Raman collect the Raman signal correspondingly. The generated image is presented in (i). For comparison, the image mapped without the terrain map is shown in (j), where the focal height is fixed during the mapping process. In (i), the mapped image is well-matched with the terrain map, on the 3D fibre surface, suggesting the improvement. However, on the bottom half of the cylinder fibre surface, the image is missed, because the laser cannot reach there to excite the signal. We thus should balance between the advantages and disadvantages of the confocal Raman imaging.

#### High resolution: TiO_2_ particles

**Fig. 5:**
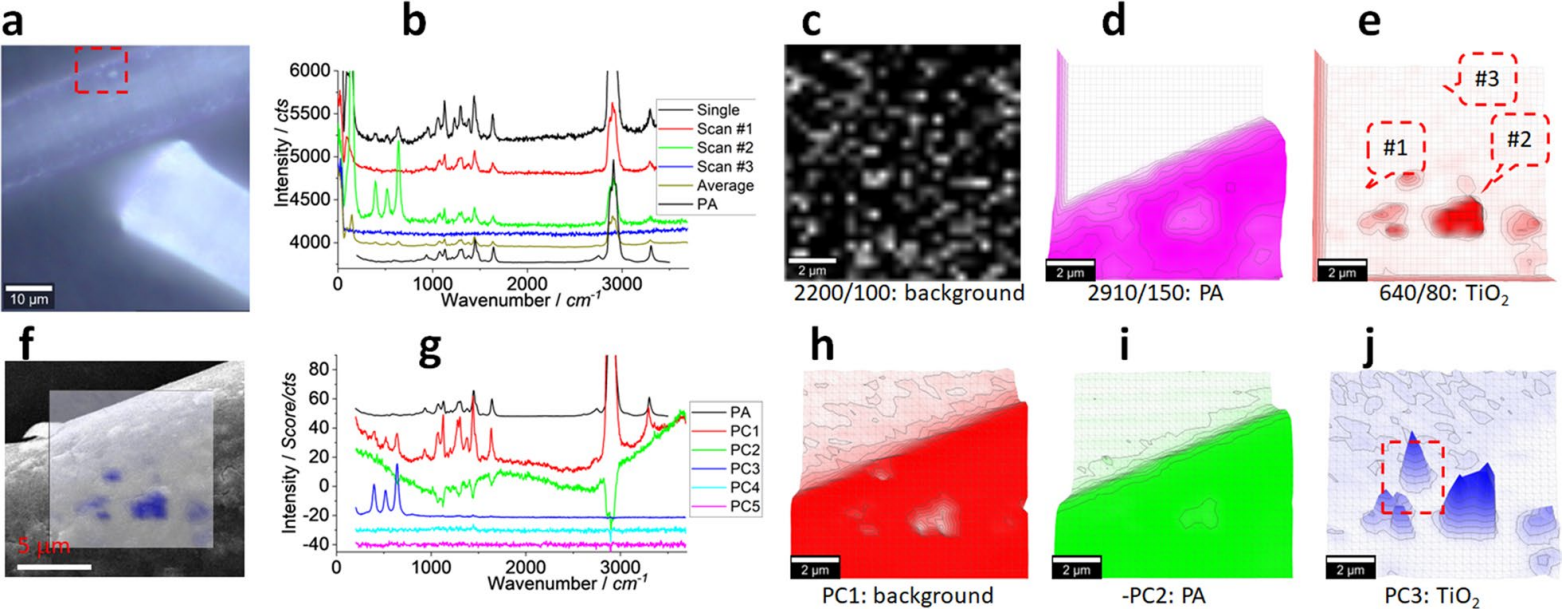
Photo image (**a**), Raman spectrum (**b**) and images (**c**–**e**), SEM image (**f**), PCA spectrum (**g**) and images (**h**–**j**). The squared area in (**a**) of 10 μm × 10 μm was scanned. Raman spectra were collected under an objective lens of 100 × , integration time of 1 s for each pixel of 0.33 μm × 0.33 μm (to create a matrix of 30 × 30). In (**b**), a single spectrum and the average spectrum of the scanning spectrum matrix are presented along with the typical scanning spectra collected from the positions marked in (**e**). In (**b**, **g**), PA’s spectrum is shown as reference. (**c**) maps a blank wavenumber window as an internal reference image. (**d**, **e**) map the PA peak at ~ 2910 cm^−1^ (**d**) and the TiO_2_ peak at ~ 640 cm.^−1^ (**e**), respectively. (**f**) merges the SEM image with the PCA image (**j**). (**g**) is PCA spectra. (**h**–**j**) map PC1–PC3, individually. In (**i**), PC2’ loading coefficient is subject to reverse. The squared area in (**j**) is further checked in Fig. 6

In this section, we zoom in to capture the details, particularly the suspected particles. We shrink the scanning pixel size to 0.33 μm × 0.33 μm, which closes to the recommended lateral scanning resolution (~ 300 nm, *λ/2NA*), but smaller than the size of the laser spot (~ 720 nm, if defined by the Airy disc, 1.22*λ/NA*) [30]. That is, in principle, the power density of the laser spot is axially descanted and follows a Gaussian surface distribution, the centroid of which has the highest power density. Once the scanning pixel shrinks, the chance for the laser centroid to scan the particle increases to emit the stronger Raman signal. The results are shown in Fig. 5.

Figure 5a shows there are particles like fish scales to coat the upper fibre surface. We focus on the upper fibre, although the signal in Fig. 3e is weaker than that on the bottom fibre. In the squared area, the typical scanning spectra are shown in Fig. 5b, collected from the positions marked in (e), along with a single spectrum and the PA spectrum. At position #2, the particle’s spectrum is dominated by three strong peaks at ~ 400 cm^−1^, ~ 520 cm^−1^ and ~ 640 cm^−1^, which are supposed to originate from TiO_2_ (anatase) [29, 32, 33]. Another peak at ~ 150 cm^−1^ is even stronger, as shown here and in the Supporting Information. These enhanced signals (stronger than these in Figs. 2–4) support above assumption that the shrink scanning pixel is helpful to visualise the details.

Before mapping the characteristic peak, we map a blank wavenumber window again, which is shown in Fig. 5c as an internal reference to check the image background. Only random noise is mapped. We then map PA as (d) and TiO_2_ as (e), using their characteristic peaks, respectively. We can see that the particles of TiO_2_ is co-formulated, echoing the SEM–EDS results in Fig. 1g, h and Additional file 1: Fig. S2. Note the image resolution is different due to the different imaging approach, including the lateral and spatial ones, as discussed above. The latter one can be related to the permeation and characterisation depth for these kinds of surface analysis. In terms of the lateral resolution, herein the Raman imaging has a scanning pixel size of 0.33 μm × 0.33 μm, much bigger than that of SEM at < 1 nm level, which might be the reason why particles are imaged at < 100 nm in Fig. 1 while 0.5–2 μm in Fig. 5. In the meantime, the EDS lateral mapping resolution (20–100 nm or higher) is also much bigger than that of SEM, which should be pointed out [34], along with the signal activity of different imaging approach.

The above solo peak’s imaging might suffer the bias and lead to a low assignment certainty. In Fig. 3, although the algebra-based algorithm is employed to catch multi peaks in the spectrum and merge them as one image, the signal beyond the selected peaks is still missed, meaning a low signal–noise ratio. A chemometrics of PCA has been recently employed to directly decode the scanning Raman spectrum matrix, for the whole set of spectrum rather than a solo peak, to participate the imaging process [21, 23]. That is, ideally, PCA can orthogonally and directly decompose the data matrix to two new matrices, one containing the spectrum profile information while another containing the intensity information. The former can generate the PCA spectrum, while the later can be mapped as image, to take all contributions from the whole set of spectrum, rather than just the several selected peaks. Using this approach, we analysis the raw data in Fig. 5’s top row and show the results in the bottom row. More details are provided in Additional file 1: Figures S5, S6.

In Fig. 5g, the PCA spectrum of PC1 is a mixture of the spectrum including the background (the non-flat baseline), the signals from fibre of PA and additive. PC2 has a reversed profile (upside down peaks) that originates from the PCA calculation. It has a non-flat baseline as well and yields the main peaks of PA, but no one of at ~ 400 cm^−1^, ~ 520 cm^−1^, ~ 640 cm^−1^. On the contrary, PC3 is dominated by these three peaks (another peak at ~ 150 cm^−1^ is shielded by the residue laser that has been deleted for PCA decoding), has a flat baseline, so that it can be directly assigned to TiO_2_ [29, 33], suggesting the advantage of PCA. The rest are assigned to the noise or the PCA calculation variation.

Once mapped as images via their loading coefficients as intensities, images in Fig. 5h–j are generated. Because PC2’s spectrum in (g) has a reversed profile, we thus minus its loading coefficient by timing (− 1). The patterns in (i, j) well match with these in (a, d, e). While (h) likes the image background, the contribution from the fibre can be identified too. (i) maps the fibre of PA, via the reversed loading coefficient or intensity. (j) clearly patterns TiO_2_ on the PA fibre surface.

Overall, these PCA images echo the assignments via the PCA spectra in Fig. 5g, suggesting the success of PCA. The advantage of imaging (area) analysis can also be evidenced by capturing the non-uniformly distributed TiO_2_ nanoparticles, which can overlap onto the SEM image and is shown as (f). It agrees with the images in Fig. 1g, h again.

While the images in Fig. 5i, j look similar with these in (d, e), the imaging and assignment certainty has been significantly increased, because the whole set of spectrum has participated the imaging process. Therefore, PCA is a suitable algorithm to analyse the scanning spectrum matrix directly. However, the result of the non-supervised analysis is heavily dependent on the data quality, which is further discussed below too.

#### Super-high resolution: TiO_2_ particles

**Fig. 6:**
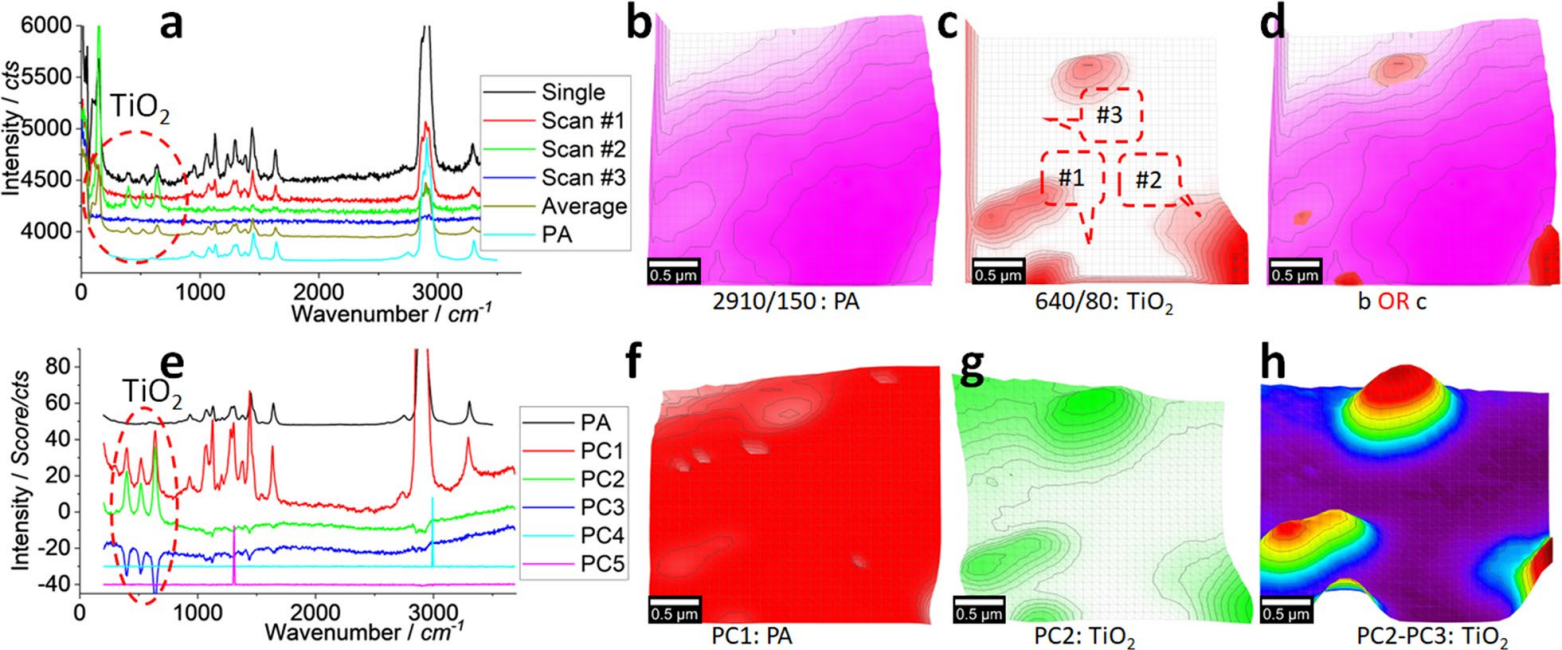
Raman spectrum (**a**) and images (**b**–**d**), PCA spectrum (**e**) and images (**f**–**h**). The squared area in Fig. 5j of 3 μm × 3 μm was scanned and Raman spectra were collected under an objective lens of 100 × , integration time of 1 s for each pixel of 0.1 μm × 0.1 μm (to create a matrix of 30 × 30). (**b**, **c**) map the characteristic peaks of PA and TiO_2_, respectively. (**d**) merges them together. (**f**–**h**) map/merge PCA intensities of PC1–PC3, as marked

We can further zoom-in the area squared in Fig. 5j, the results are presented in Fig. 6. The size of the scanning pixel (100 nm × 100 nm) is smaller than the recommended one at 300 nm. As said, by shrinking the scanning pixel size, we hopefully capture more details via an increased signal–noise ratio that is expected to be emitted by the centroid of the laser spot [13, 30]. Even so, it is not expected to get the exactly matched image between SEM–EDS and Raman, as presented in Figs. 1h and 5f, rather than to benefit each other. For example, SEM can provide the morphology information, while Raman can identify the target from molecular spectrum perspective.

In Fig. 6a, the typical spectra are shown again, along with a single spectrum (with an integration time 10 s *vs.* 1 s) and the standard spectrum of PA as a reference. Basically, the situation is similar with that in Fig. 5, we can suspect the presence of PA and TiO_2_.

Once mapped to visualise them, images in Fig. 6b–d are generated. Again, we can see the nanoparticles of TiO_2_ “decorate” the surface of the PA fibre, which can be clearly observed in (d), once merged. Note that the different peak intensity (of PA and TiO_2_, as pseudo *z*-axis) means the non-discriminated merge might shield some pattern details by each other or lead to the bias image, which has been discussed above. More Raman images are provided in Additional file 1: Figs. S7, S8.

Again, we employ PCA to extract their signals, and the results are shown in Fig. 6e–h. In (e), the PCA spectra are different from those in Fig. 5g. As said, the non-supervised PCA is heavily dependent on the data quality. Although the PC1 spectrum that is similar with that in Fig. 5g, including the background of a non-flat baseline and the contributions from PA and TiO_2_, PC2 and PC3 are different. PC2 is dominated by TiO_2_, due to the appearance of peaks at ~ 400 cm^−1^, ~ 520 cm^−1^ and ~ 640 cm^−1^ [29, 33]. However, the non-flat baseline means some background information is not completely separated. PC3 has a reversed profile and seemingly dominated by TiO_2_ as well.

Once their loading coefficients are mapped, images in Fig. 6f, g are generated, for PC1–PC2. (f, g) are similar with images (b, c), suggesting the success of PCA again. Because PC3 also contains the TiO_2_ peaks (circulated in (e), due to the non-supervised PCA), we can intentionally extract and pick up all of the contributions from different PC via algebra-based algorithm towards merge, as shown in (h), to better visualise TiO_2_. Herein, PC3’s loading coefficient or pseudo-intensity has been reversed by timing (− 1) to merge with PC2. In brief, the presence of TiO_2_ can be confirmed. More details and discussion on the PCA-algebra algorithm are provided in Additional file 1: Fig. S8.

#### Fibre counting and other swabs

The estimate on the fibre releasing amount is conducted in Additional file 1: Fig. S9, which is 5–100 fibres from a mimicked sampling (from 10 mimicked samplings) and might be a concern [35]. More research is needed to understand the risk assessment. We should clean and wash our nose, mouth after the sampling, if facilities are available.

We check more brands of swab in Additional file 1: Figs. S10–S16, which are summarised in Table 1 and Additional file 1: Figure S10b. Basically, all swab tips are made of plastics, either PA or others, with configuration of brush or sponge. In general, swabs with sponge heads release almost no debris during the sampling process, while brush swabs are more likely to shed fibres as microplastics. All fibres checked herein are accompanied with the TiO_2_ additive [26, 27]. Beyond as a universal pigment, TiO_2_ is added for several possible reasons. TiO_2_ has antibacterial properties that can inhibit the growth of bacteria on synthetic fibres [36]. This is particularly important for medical applications. Adding TiO_2_ to synthetic fibres can also improve UV protection, thermal stability and flame retardancy, and thus increase their durability and extent their life span [28, 37]. No cotton swab is found herein.

**Table 1:** Summary of the checking results

Sample #	Tip configuration	Plastic type	TiO_2_ particle presence	Microplastic release amount, per sampling
#1	Brush	PA	Yes	~ 44
#2	Sponge	PUR, PA	No	0
#3	Brush	PA	Yes	~ 12
#4	Brush	PA	Yes	~ 33
#5	Sponge	PUR, PA	No	0
#6	Sponge	PUR, PA	No	0
#7	Brush	PA	Yes	~ 29

The amount of the released fibres may be affected by several factors such as the quality of the material used to make the swab, the technique used to manufacture the swab, and the method of use (e.g., sampling skills), saliva viscosity and other conditions. The degree of shedding can be worse if the swab is not of good quality or is not handled properly. To minimize the risk of fibre shedding, it is important to use suitable swabs that are designed specifically for certain applications (e.g., COVID-19 sampling and others). From the microplastic and nanoparticle’s point of view, the sponge swab or the cotton stick is recommended. Swabs should also be handled carefully, with minimal rubbing or bending, to prevent fibres from coming loose. Previous research has reported that accidental hard contact with nasal anatomical structures can promote the fragmentation of swabs and lead to release of more fibres [38].

## Conclusions and remarks

There is a big concern on the COVID-19-related plastic waste. Taking the disposal self-testing kit as an example, a kit is ~ 10 g in weight and most of the components are made of plastics, including package, tube, swab, container, cartridge etc. In China, for example, the population is ~ 1.4 billion. During the COVID-19 season, almost everyone has been tested for 10–100 times, meaning 14–140 billion of kits have been used for sampling. Globally, the amount can be significant. The results herein send us a strong warning that we should be cautious with the testing kit, from microplastic point of view.

Regarding the COVID-19-related plastics waste, while the aftermath treatment (e.g., incineration) can hopefully solve the potential environmental contamination issue, the testing kit can directly deliver some un-expected aliens into our nose or mouth during the sampling process. As shown herein, if the swab is made of plastic fibres at the tip, it is highly likely that the microplastics can be left behind, along with the additive, such as TiO_2_ particles. If we concern the potential microplastics issue, we should wash our nose and clean our mouth after each sampling, and we should select suitable swab, such as with configuration of sponge, rather than with configuration of brush. It should be noted that the results from this study may not reflect the real-world situations, as the material (glass, aluminium and carbon tape) used to collect fibres is different from human nasal tissues. It is thus difficult to estimate the exact amount of fibres that can potentially be inhaled or ingested by a human after COVID testing. Further research is required to better assess potential human exposure to microplastics resulting from swab sampling, so that regulators and manufacturers can be aware of the risks and take appropriate measures towards the safer practices.

Raman imaging can be effectively employed to characterise the microplastics and the accompanied additives, particularly when the target distribution is not uniform. The single spectrum (or point analysis) can easily bypass and miss the detail, generating the result bias. The scanning process (area/imaging analysis) to generate image can pick up all these non-uniform distributions and directly visualise them. The possible false image via the confocal Raman should also be noted. More research is needed to further validate this characterisation approach.

## Supplementary Information

**Additional file 1: Figure S1.** Photo images. **Figure S2-1/3.** More EDS analysis for Fig. 1. **Figures S3–S4.** Swab bar and testing cartridge identification for Sample #1. **Figure S5.** More Raman images for Fig. 5. **Figure S6.** More PCA parameters for Fig. 5. **Figures S7–S8.** More Raman images and PCA parameters for Fig. 6. **Figure S9.** Fibre counting. **Figure S10.** More swab Samples. **Figure S11.** Nasal swab tip, Sample #2. **Figure S12.** Nasal swab tip, Sample #3. **Figure S13.** Nasal swab tip, Sample #4. **Figure S14.** Nasal swab tip, Sample #5. **Figure S15.** Nasal swab tip, Sample #6. **Figure S16.** Nasal swab tip, Sample #7.

## Data Availability

All data generated or analysed during this study are included in this published article [and its Additional files].
