# Supplementary material for: Identification of microplastic fibres released from COVID-19 test swabs with Raman imaging
Source: Environ Sci Eur. 2023 May 6;35(1):34. doi: 10.1186/s12302-023-00737-0 (PMC10162899; doi:10.1186/s12302-023-00737-0)
Supplement: Supplementary file 1 — Additional file 1: Figure S1. Photo images. Figure S2-1/3. More EDS analysis for Fig. 1. Figures S3–S4. Swab bar and testing cartridge identification for Sample #1. Figure S5. More Raman images for Fig. 5. Figure S6. More PCA parameters for Fig. 5. Figures S7–S8. More Raman images and PCA parameters for Fig. 6. Figure S9. Fibre counting. Figure S10. More swab Samples. Figure S11. Nasal swab tip, Sample #2. Figure S12. Nasal swab tip, Sample #3. Figure S13. Nasal swab tip, Sample #4. Figure S14. Nasal swab tip, Sample #5. Figure S15. Nasal swab tip, Sample #6. Figure S16. Nasal swab tip, Sample #7. [file 12302_2023_737_MOESM1_ESM.docx]

Identification of microplastic fibres released from COVID-19 test swabs with Raman imaging

Cheng Fang^a, b^*, Yunlong Luo^a,b^, Clarence Chuah^c^, Ravi Naidu^a, b^

^a^Global Centre for Environmental Remediation (GCER), University of Newcastle, Callaghan NSW 2308, Australia.

^b^Cooperative Research Centre for Contamination Assessment and Remediation of the Environment (CRC CARE), University of Newcastle, Callaghan NSW 2308, Australia.

^c^Flinders Institute for NanoScale Science and Technology, College of Science and Engineering, Flinders University, South Australia 5042, Australia.

*Corresponding author: Cheng Fang, Tel: +61 2 4913 8740; Fax: +61 2 4913 8740; E-mail: [cheng.fang@newcastle.edu.au](mailto:cheng.fang@newcastle.edu.au). <https://orcid.org/0000-0002-3526-6613>

***Supporting Information***

Contents

[1. Figure S1: photo images 3](#_Toc131147426)

[2. Figure S2-1/3: more EDS analysis for Figure 1 4](#_Toc131147427)

[3. Figures S3-S4: swab bar and testing cartridge identification for Sample #1 6](#_Toc131147428)

[4. Figure S5: more Raman images for Figure 5 7](#_Toc131147429)

[5. Figure S6: more PCA parameters for Figure 5 8](#_Toc131147430)

[6. Figures S7-S8: more Raman images and PCA parameters for Figure 6 9](#_Toc131147431)

[7. Figure S9: fibre counting 12](#_Toc131147432)

[8. Figure S10: more swab samples 13](#_Toc131147433)

[9. Figure S11: nasal swab tip, Sample #2 14](#_Toc131147434)

[10. Figure S12: nasal swab tip, Sample #3 15](#_Toc131147435)

[11. Figure S13: nasal swab tip, Sample #4 16](#_Toc131147436)

[12. Figure S14: nasal swab tip, Sample #5 16](#_Toc131147437)

[13. Figure S15: nasal swab tip, Sample #6 17](#_Toc131147438)

[14. Figure S16: nasal swab tip, Sample #7 18](#_Toc131147439)

[References 19](#_Toc131147440)

## Figure S1: photo images


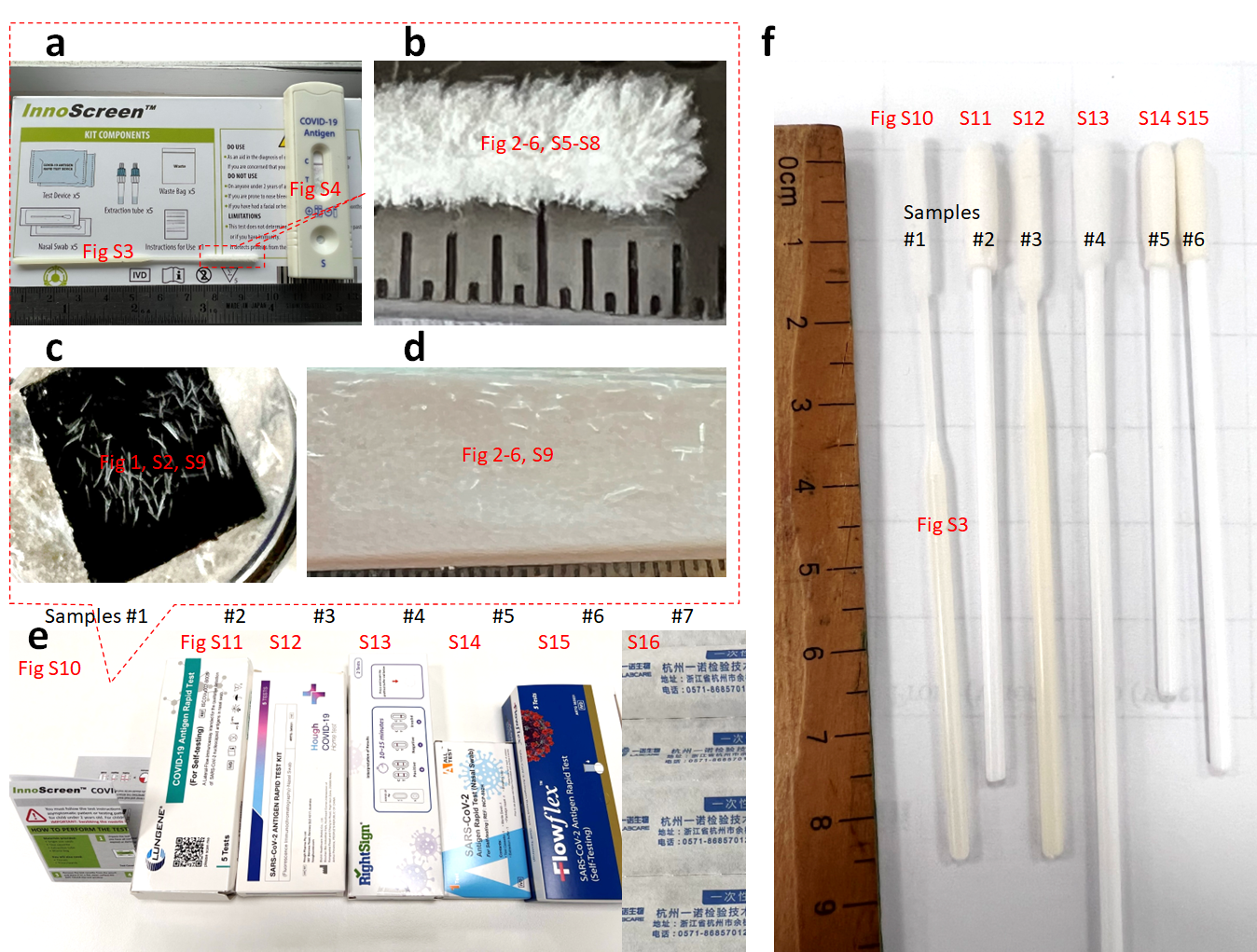


Figure S1. Photo images of the samples. (a-d) shows details for Sample #1, which is among the list in (e) and focused in this study. (a) shows the package box, the testing cartridge and the nasal swab. (b) zooms in the swab tip. (c) shows the released fibres on the carbon tape surface. (d) demonstrates the released fibres on the glass slide surface. The minimum scale in the ruler in (b, d) is 0.5 mm. (e) lists the 7 samples we checked, (f) presents their swabs. The figures to present the results are suggested.

The samples we have collected are shown here, with details and the brand information. Sample #1 is focused first and the swab tip configuration is shown in (b). The released fibres can be visualised in (c, d), which give us a concern and is the reason why we check. Other samples are listed in (e) and also checked, by expanding the results from Sample #1, and focusing on the swab tips presented in (f). Sample #7 was used in China and tested as a reference for comparison, which is for the subsequent PCR test, different from others for antigen test.

## Figure S2-1/3: more EDS analysis for Figure 1


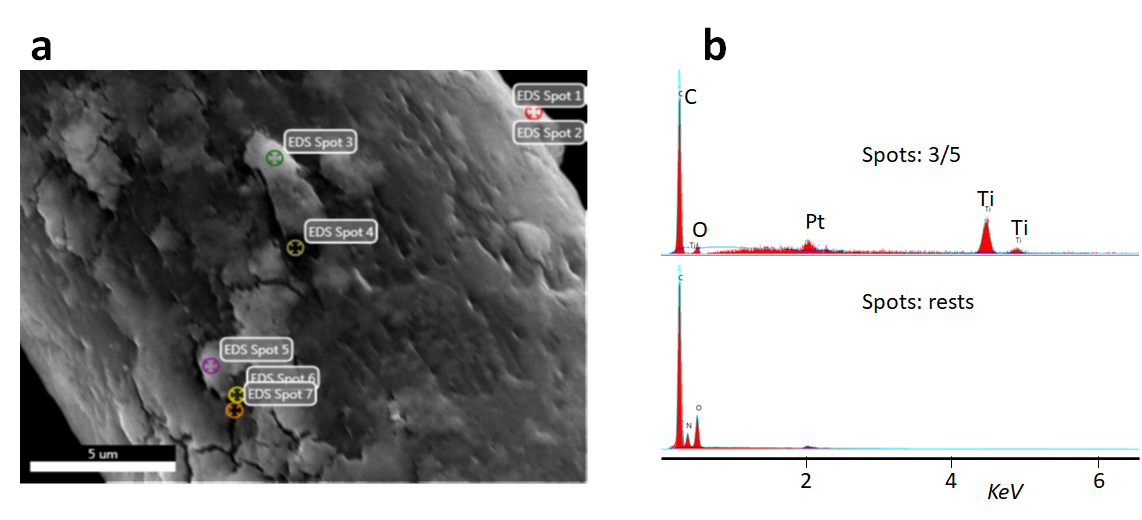


Figure S2-1. SEM image (a) and the typical EDS (b). They are collected from the swab tip for Sample #1 and the collection positions are marked in (a).

The EDS suggests the presence of titanium (Ti), for spots 3/5, but not for every spot. The peak of Pt should be ignored because it originates from the sputter-coating. Ti is not uniformly distributed, as discussed in Figure 1. This is the reason why we need scan the sample in order to capture it, no matter for EDS (element analysis) or for Raman (molecule analysis). That is, area analysis or imaging is recommended rather than point analysis.


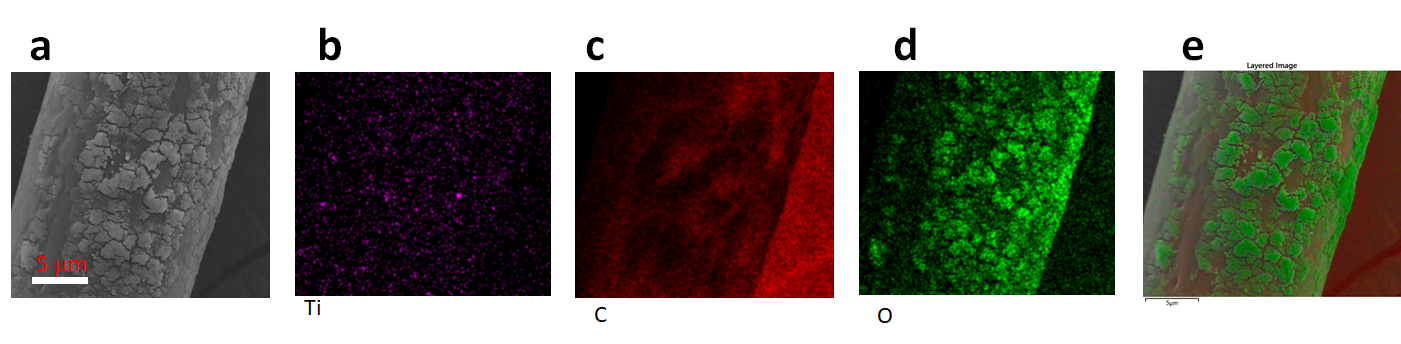


Figure S2-2. SEM image (a) and EDS mapping images (b-e) for Figure 1(d). The element is suggested on the bottom. (e) merges them together.

By scanning the sample, the element mapping images are provided here. Ti has a weak (due to the low amount, <3%) and non-uniform distribution along the fibre where others are strong. (c) is dominated by the background carbon tape.


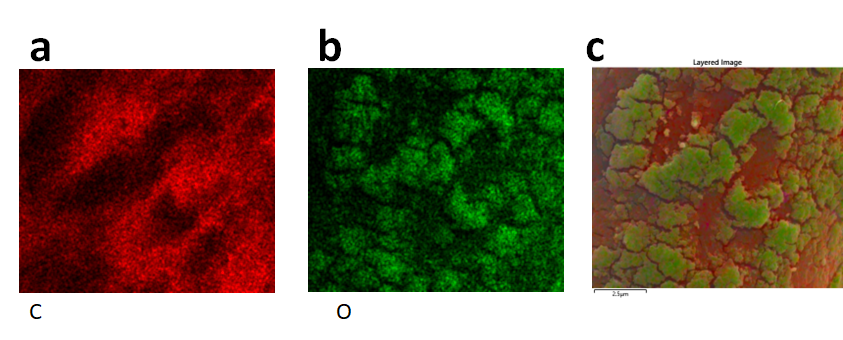


Figure S2-3. More EDS mapping images for Figure 1(h).

Once zoomed in, more EDS images are provided here for Figure 1(h), to visualise the distributions of different elements including carbon and oxygen. (c) overlaps them onto the SEM image.

## Figures S3-S4: swab bar and testing cartridge identification for Sample #1


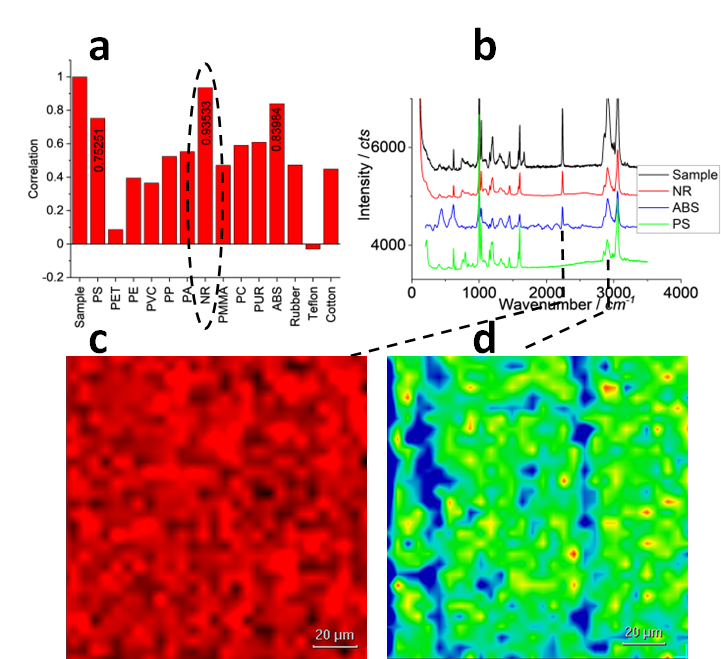


Figure S3. Swab bar identification, including correlation value (a), Raman spectrum (b) and images (c, d) mapping the characteristic peaks, as suggested, for Sample #1.

The nasal swab has a tip and a bar. The bar material is identified here, to be NR or mixture of PS or ABS. The mapped images can suggest the presence of organics or plastic.


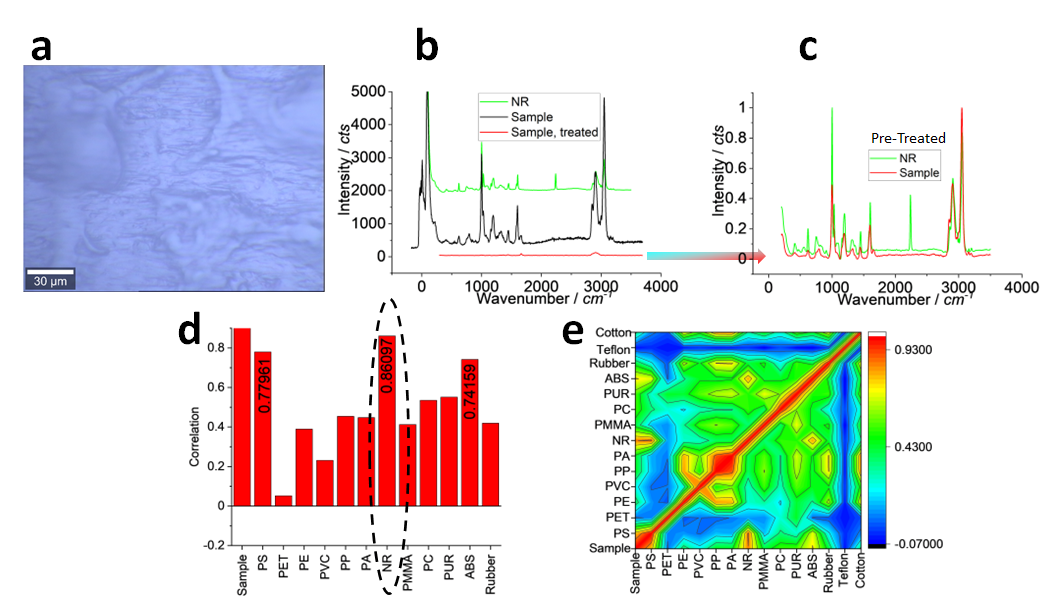


Figure S4. Cartridge identification, including photo images (a), Raman spectrum (b, c), correlation value (d) and matrix (e), for the testing cartridge in Sample #1.

The cartridge is identified as NR as well (or dominated by NR if it is a mixture), for Sample #1, via the similar analysis conducted in Figure 2. However, at different positions, the materials might be different, in order to realise the designed function.

## Figure S5: more Raman images for Figure 5


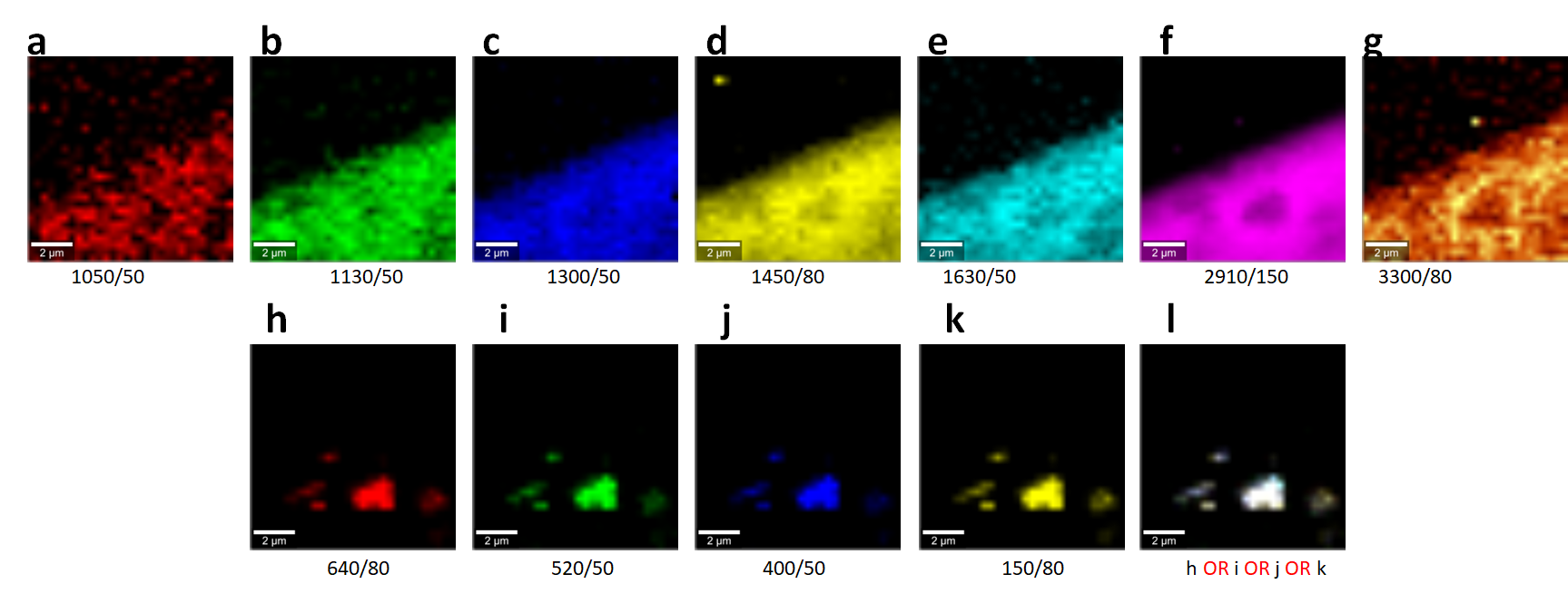


Figure S5. Raman images mapping the characteristic peaks of PA (top row) or TiO_2_ (bottom).

More Raman images are mapped here to visualise the PA (top row) and TiO_2_ (bottom row). They are similar and suggest the presence of PA and TiO_2_. Particularly (l) merges four images to further confirm the presence of TiO_2_.

## Figure S6: more PCA parameters for Figure 5


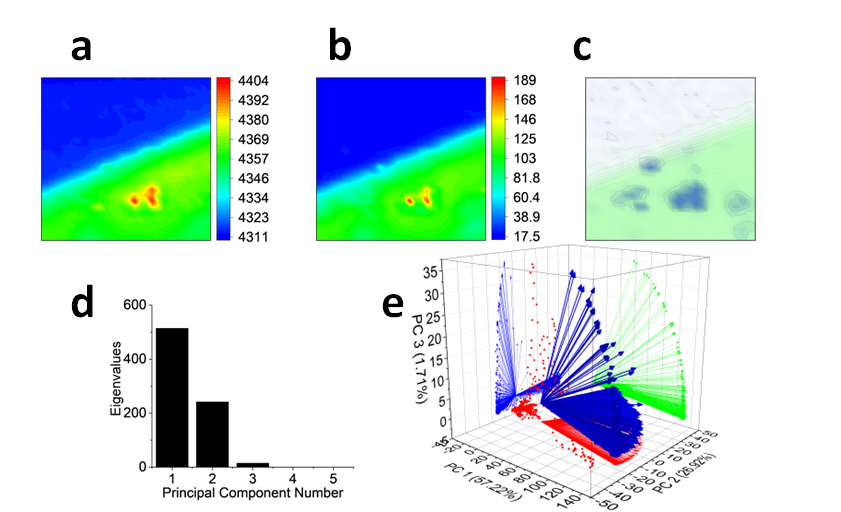


Figure S6. PCA parameters. (a) maps the mean, (b) maps the standard deviation, (c) maps PC2 and PC3 together, (d) is the scree plot and (e) is the bi-plot.

The PCA analysis parameters are shown here. The mapped mean in (a) can tell us where the signal is strong or the main eigenvalue variance area, while the mapped deviation in (b) reminds us where the variation is big and we should pay more attention. (c) merges PC2 and PC3 to compare them. The scree plot in (d) suggests the main variance of the eigenvalues has been taken by PC1-PC3. (e) suggests the main PCs are almost independent from each other, which is the nature of the PCA analysis.

## Figures S7-S8: more Raman images and PCA parameters for Figure 6


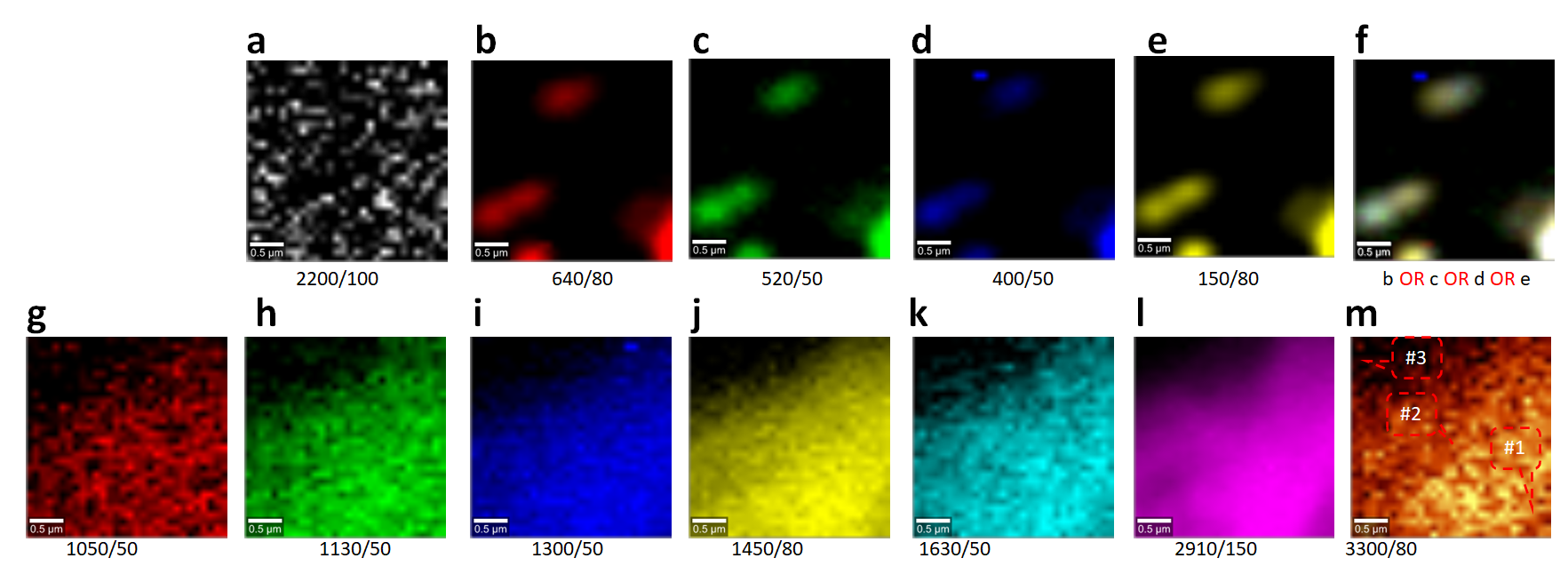


Figure S7. Raman images mapping the characteristic peaks of TiO_2_ (top row) or PA (bottom).

More Raman images are provided. The top row can confirm the presence of TiO_2_ while the bottom row can confirm the presence of PA, with an internal refence image in (a).


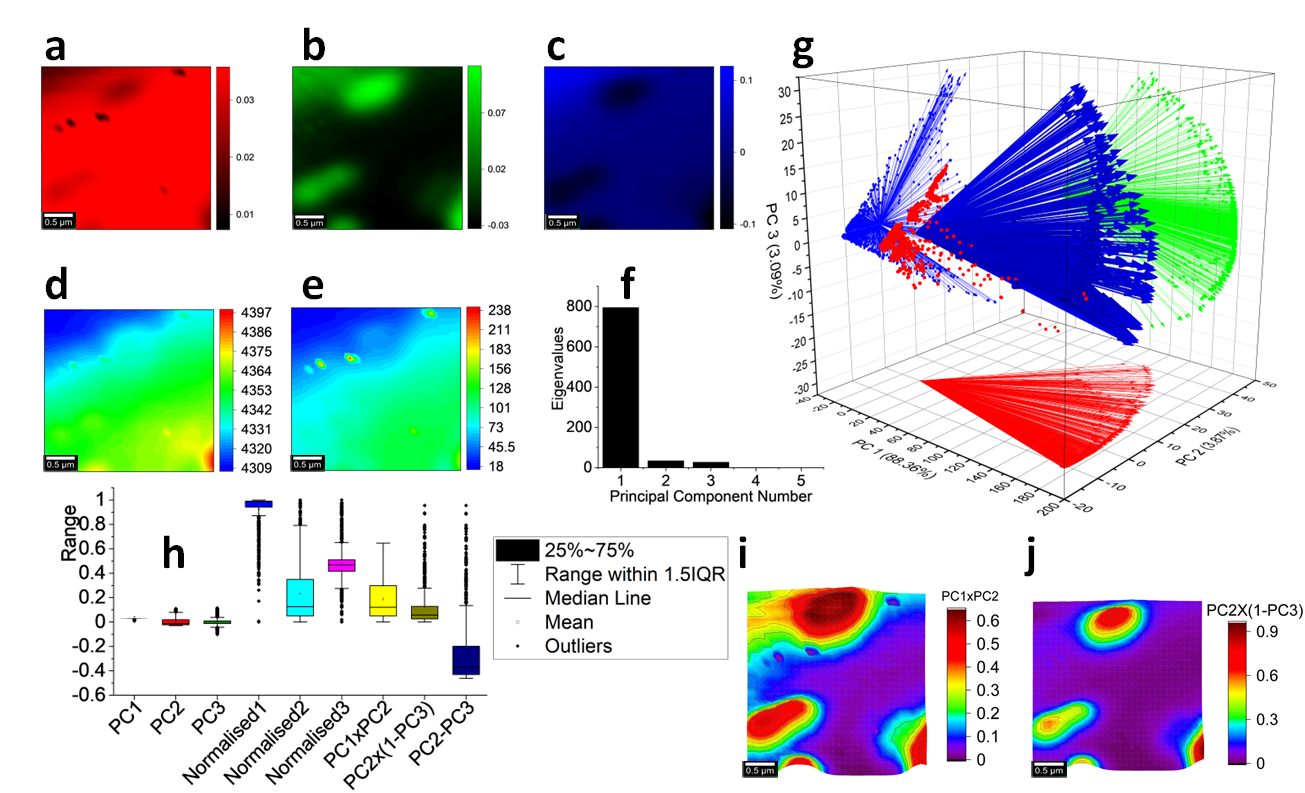


Figure S8. PCA parameters. (a-c) map PC1-PC3, respectively. (d) maps the mean, (e) maps the standard deviation, (f) is the scree plot, (g) is the bi-plot, (h) lists the distribution ranges of the loading coefficients, before / after normalisation and merge, (i, j) are the merged images, as indicated by the colour scale bars.

The PCA parameters are provided here, which are similar with these in Figure S6, including the mapped mean / deviations (d, e), scree plot (f) and bi-plots (g). Furthermore, the individual PCs’ loading coefficients are mapped in (a-c) as well. As discussed in the main manuscript, from the PCA spectra, both PC1 and PC3 contain some information of TiO_2_. In this case, we can intentionally pick up their contributions and merge with PC2, as shown in (i, j). Using the algebra-based algorithm, we can effectively correct the non-supervised PCA calculation.

To this end, we need first normalise the loading coefficients or the pseudo-intensities to 0-1, to avoid bias. That is, the PCA generates the PCs to take the eigenvalue variances at different percentage, 88.36% for PC1, 3.87% for PC2, 3.09% for PC3, 0.20% for PC4 and 0.18% for PC5, respectively. While these percentages can be employed to suggest the contribution even the target size (Cheng et al., 2022), the direct merge can meet the bias, as shown in (h). After being normalised to 0-1, the timing/multiplying can pick up the higher parts (closing to 1) more effectively. Consequently, the merged images (i, j) are produced, echoing the results in Figure 6 to confirm the presence of TiO_2_.

## Figure S9: fibre counting


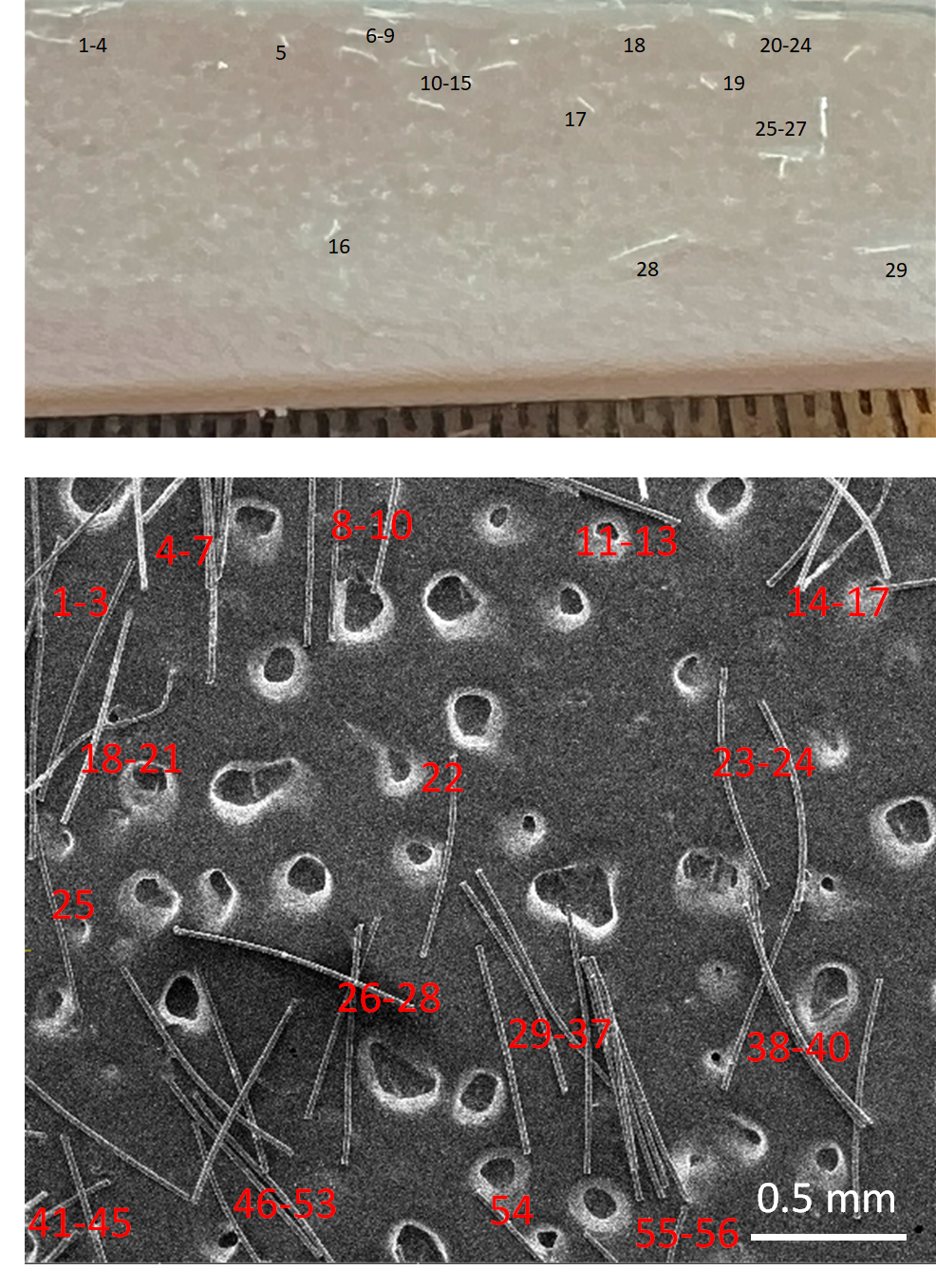


Figure S9. Fibre counting via photo image (top) and SEM image (bottom).

The counting of the released fibres is conducted here, with the sequence number marked, in the photo image and SEM image. Basically 5-100 fibres (from 10 mimicked samplings) can be counted. Note those images are just parts for the whole pictures, to show the majority parts that contains most of the released fibres.

The counting on particles of TiO_2_ is difficult. In the main manuscript, we can see that each fibre might contain hundreds or thousands of TiO_2_ particles, if they are uniformly distributed on the fibre surface. Unfortunately, the distribution is not uniform, as evidenced in this study.

The test on Sample #1 will be extended to other brands’ of testing kits, as presented below.

## Figure S10: more swab samples


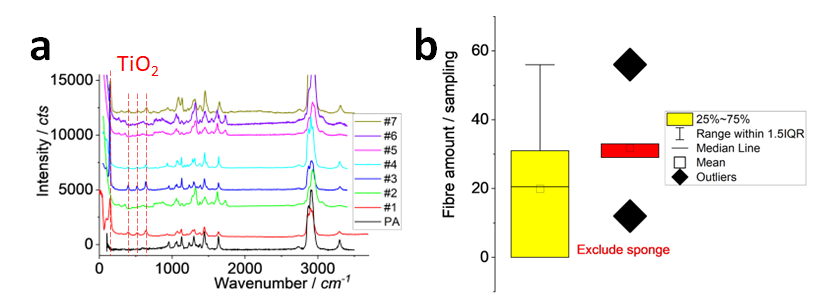


Figure S10. Raman spectra of different samples (a) and the statistical analysis on the amount of the released fibres (b).

The typical Raman spectra of different samples are compared in (a). Basically, PA can be confirmed for samples #1, #3, #4 and #7, along with the presence of TiO_2_ (as marked by the dashed lines). At their tips of the nasal swab, the configurations are brush. Samples #2, #5 and #6 are suspected to be PUR or some mixtures that are not clear so far, which are sponge at the swab tip. The further discussion is provided below.

In (b), we can see the releasing amount. If we exclude the sponge configuration, ~30 fibres/sampling is estimated to be released. This estimation has variation that is discussed in the main manuscript.

## Figure S11: nasal swab tip, Sample #2


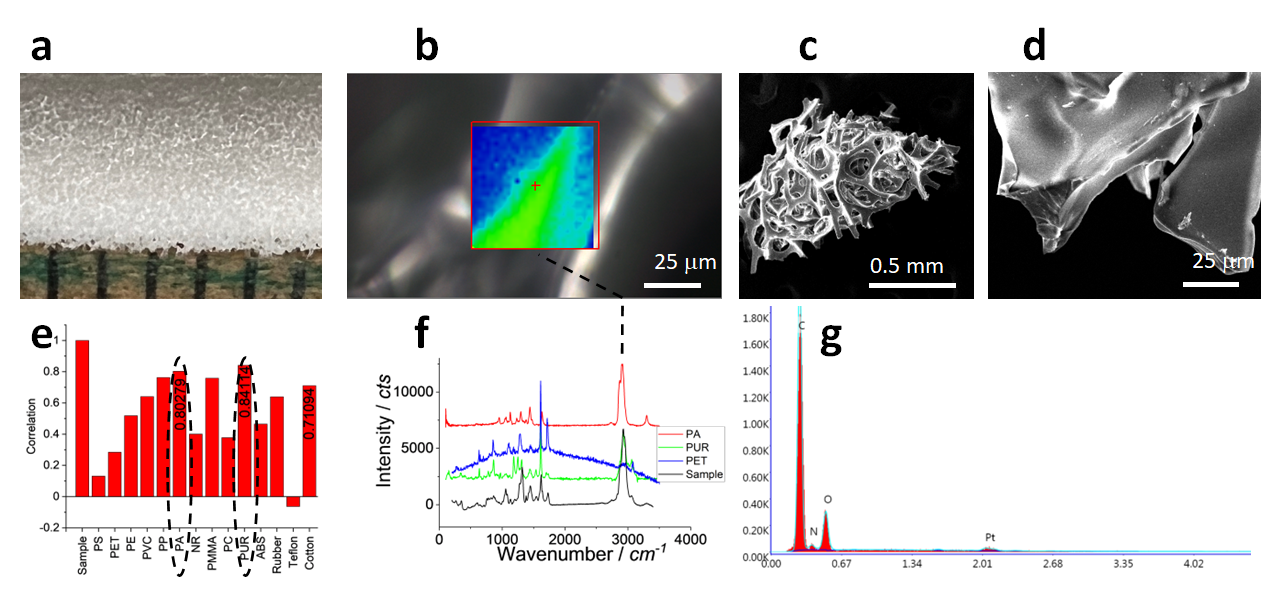


Figure S11. Analysis on Sample #2, swab tip. (a) shows the photo image of sponge. (b) is the Raman image overlapped on the photo image. (c, d) are SEM images. (e) lists the correlation values. (f) shows the typical spectrum to compare with PA, PUE and PET. (g) is the typical EDS spectrum.

The analysis on the swab tip of Sample #2 is provided here. (a) shows the sponge configuration that is different from the brush shown in Figure S1(b), which can be supported by the SEM images in (c, d). The Raman image in (b) can suggest the presence of the plastic, where the focal height’s effect can be observed again, because only the focused grid bridge can be effectively imaged. The suspected items are suggested in (e), might be PA, PUR or others, or their mixture. (f) compares its typical spectrum with several standard spectra.

The good thing is the mimicked sampling does not release significant amount of debris from this swab tip, and also no particle of TiO_2_ released, as evidenced by the absence of Ti in (g). That is, the sponge structure of grid is stable and can effectively avoid the release of debris. The test here is thus conducted on the sponge surface, which is different from these in the main manuscript that are conducted on the released fibre’s surface.

## Figure S12: nasal swab tip, Sample #3


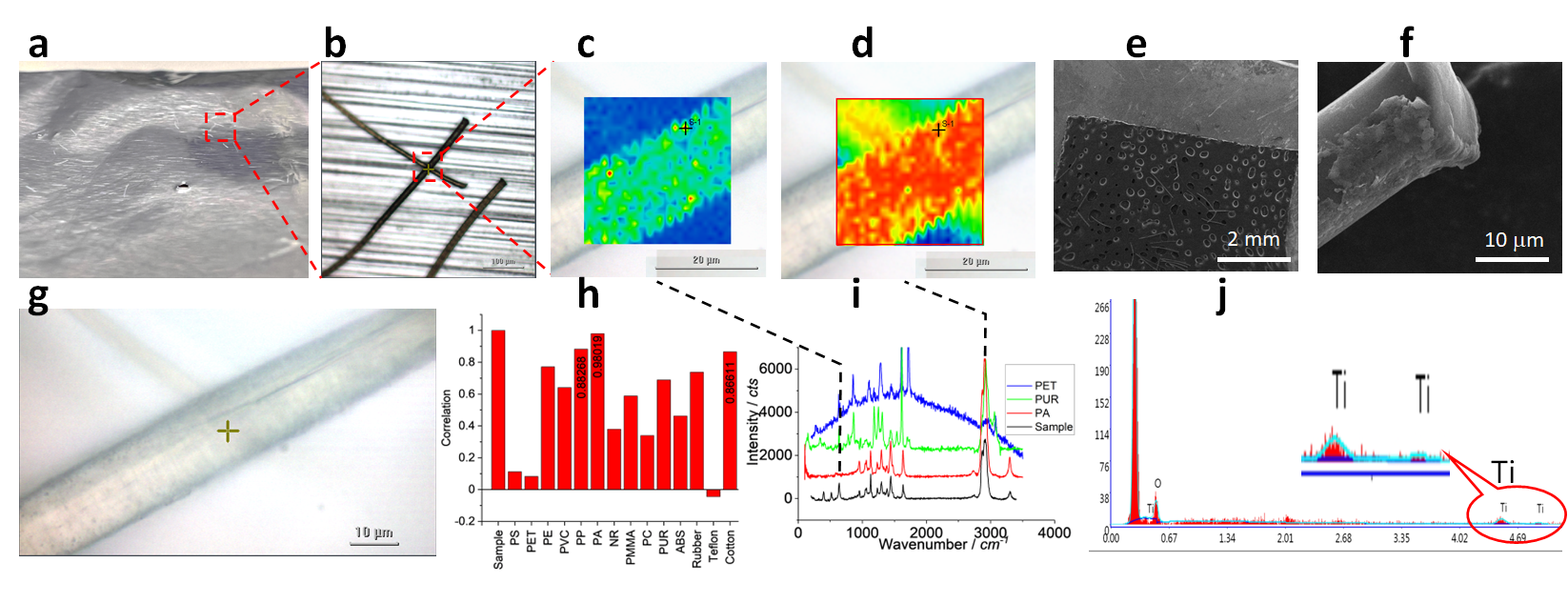


Figure S12. Analysis on Sample #3, swab tip. (a, b, g) show the photo images, and (e, f) are SEM images, at different magnifications. (c, d) map the typical peaks indicated in the Raman spectra in (i). (h) lists the correction values. (j) is the typical EDS spectrum.

Sample #3 yields the similar results with #1 listed in the main manuscript. The released fibres are visualised in (a, b, g) on the aluminium foil surface. Once zoomed in and scanned, images (c, d) are generated. The mapping images of TiO_2_ (c) and PA (d) are also similar with Sample #1, to confirm their assignments, as suggested by (h, i). (e, f) are SEM images that are similar with these in the main manuscript: the released fibres (e) and the coating on the fibre surface (f). The presence of Ti can be confirmed by the EDS in (j), as marked.

## Figure S13: nasal swab tip, Sample #4


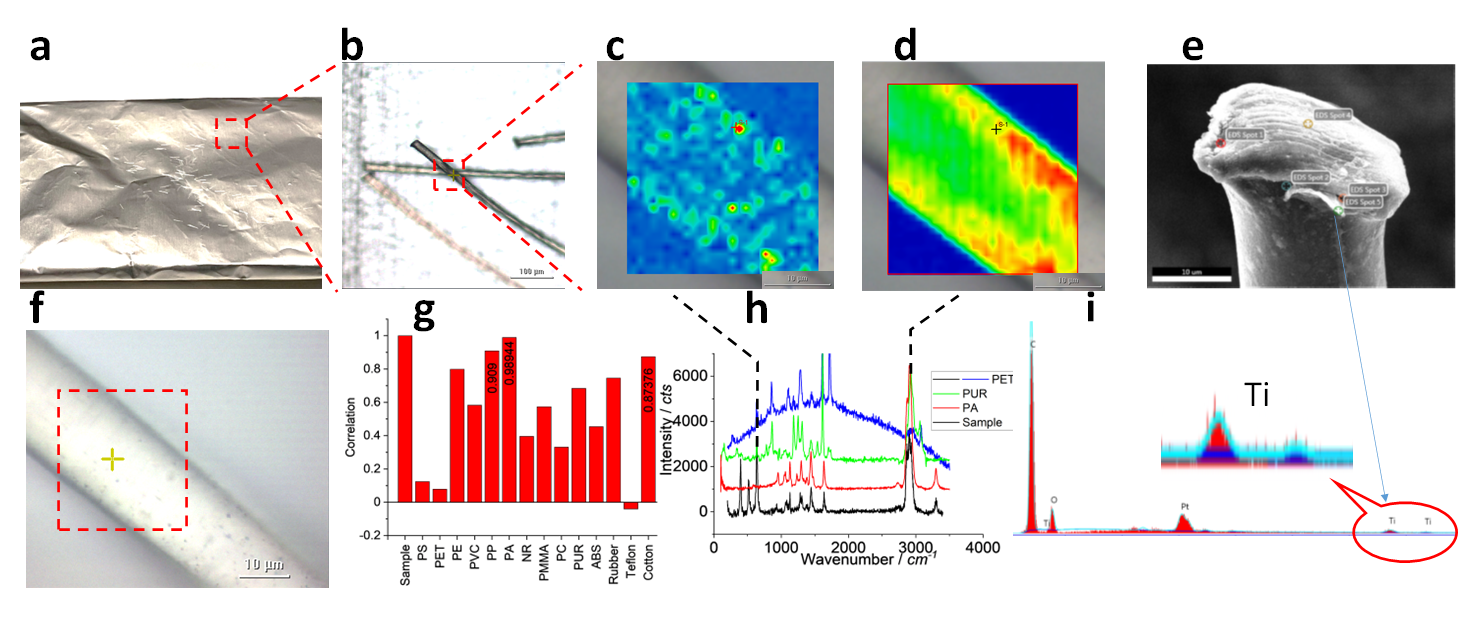


Figure S13. Analysis on Sample #4, swab tip. (a, b, f) show the photo images at different magnifications. (c, d) map the typical peaks indicated in (h). (g) lists the correction values. (e) is a SEM image with position marked to collect EDS presented in (i).

For this sample, the similar results are gotten as above, PA fibres can be released. TiO_2_ can be visualised in (c) by mapping its characteristic peak, and evidenced by EDS in (i), particularly from the zoomed-in area in the inset.

## Figure S14: nasal swab tip, Sample #5


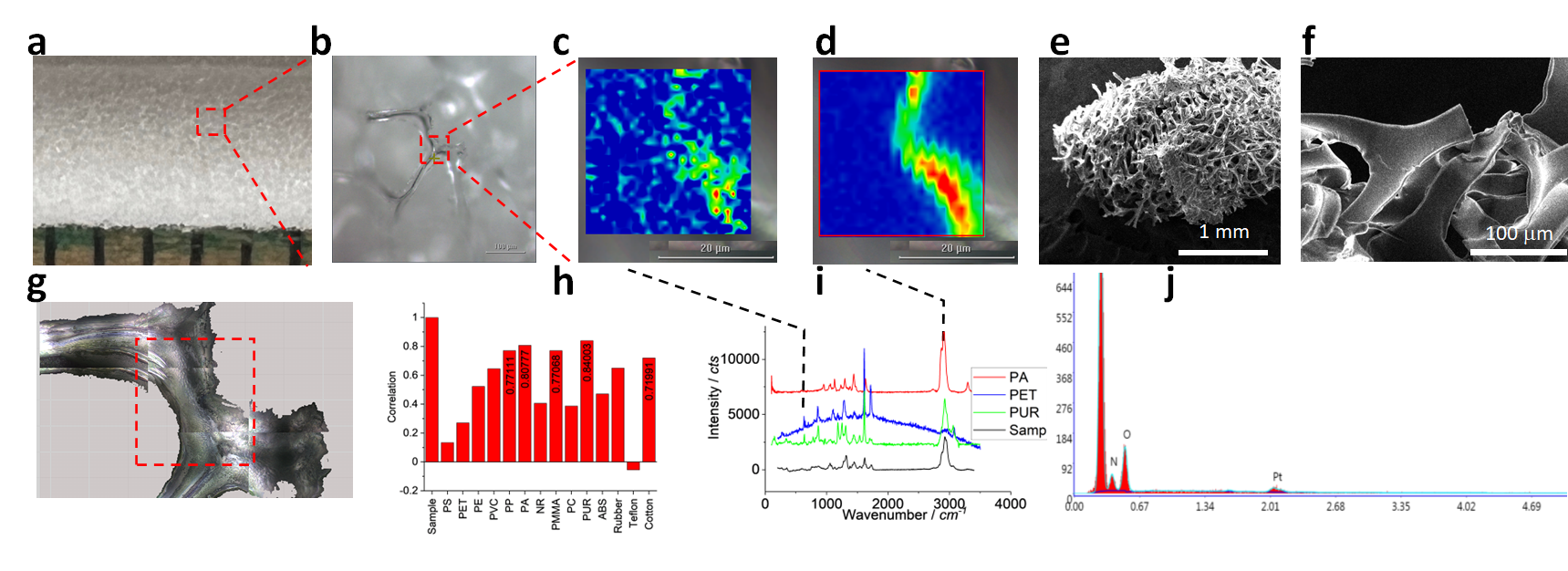


Figure S14. Analysis on Sample #5, swab tip. (a, b, g) show the photo images and (e, f) present the SEM images at different magnifications. (g) is the terrain map of the surface. (c, d) map the typical peaks indicated in (i). (h) lists the correction values and (j) is the EDS.

For this sample, the similar results are gotten as Sample #2, the sponge of the swab tip might be PA, PUR or others, or mixture. The grid structure of the sponge can be visualised in (a, b, e-g), where (g) is the terrain map, collected by changing the focusing height along *z*-axis. The confocal Raman images in (c, d) can suggest the presence plastics. (j) suggests that the presence of Ti is ignorable.

## Figure S15: nasal swab tip, Sample #6


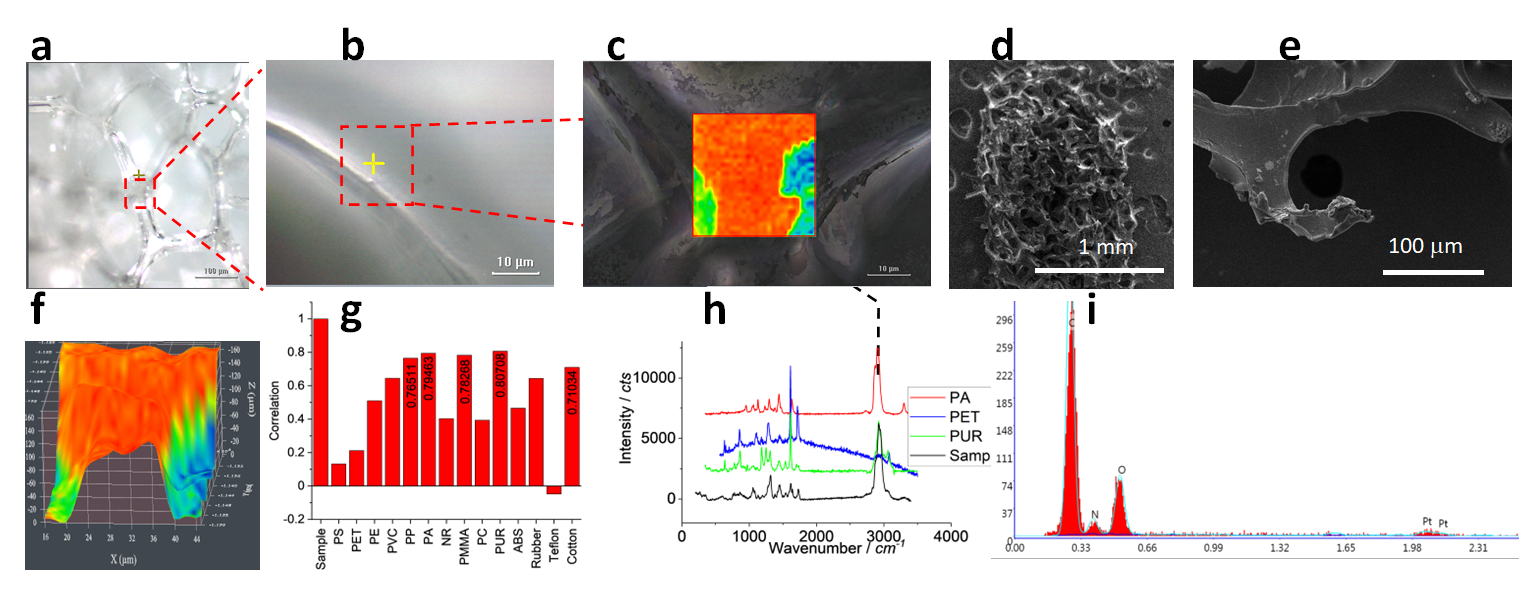


Figure S15. Analysis on Sample #6, swab tip. (a, b) show the photo images and (d, e) are the SEM images at different magnifications. (c, f) map the typical peak indicated in (h), while (f) shows the terrain Raman image collected according to the terrain map, by change the focusing height. (g) lists the correction values and (i) is the typical EDS spectrum.

For Sample #6, the similar results are gotten as above Sample #5, the sponge is plastic but does not release obvious debris, during the mimicked sampling process. Herein the test was conducted on the sponge surface again that has been cut by a stainless scissors. The focal height’s effect is demonstrated in (f), where the terrain imaging is achieved via the terrain map.

## Figure S16: nasal swab tip, Sample #7


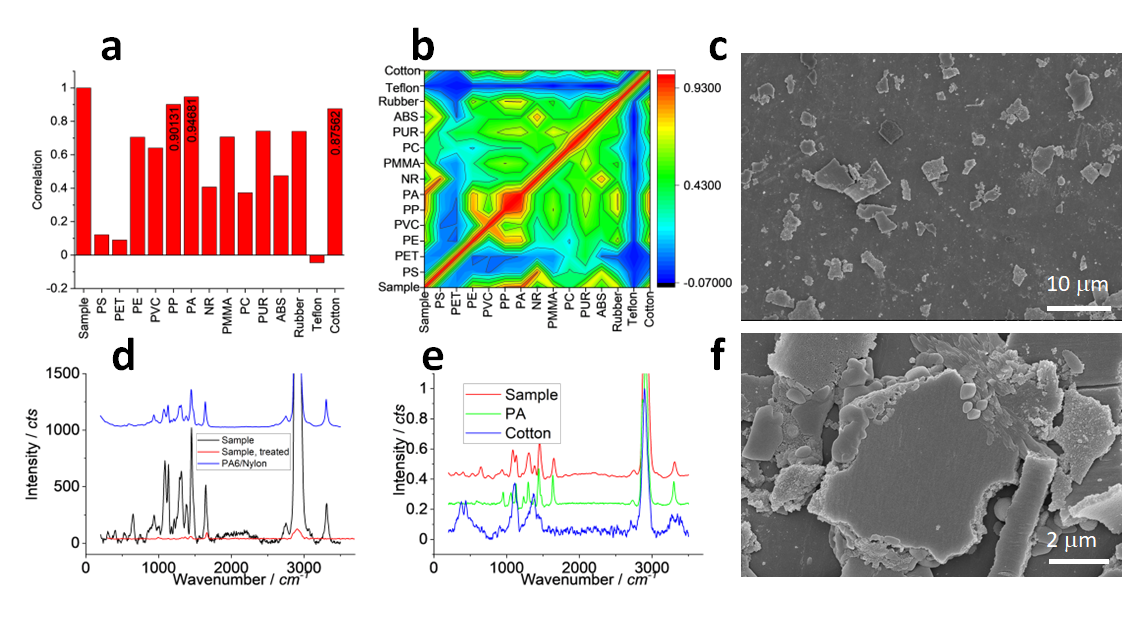


Figure S16. Analysis on Sample #7, swab tip and debris. (a) lists the correction values, (b) is the correlation matrix, (d, e) show the Raman spectrum, (c, f) are SEM images to show the debris released from the swab tip.

Sample #7’s swab tip is mainly made of PA again. For this assignment in (a, b), the spectrum has been pre-treated, as demonstrated in (d, e). In (e), the comparison between the sample’s spectrum and the standard spectrum of PA is provided, along with the spectrum of cotton.

Along with the released fibres that were analysed in (a-e), the released and left-behind debris are also observed and presented in (c, f), which is generated by vigorously tapping the swab tip onto the glass surface. These debris might be also released during the sampling process too, no matter for the subsequent antigen test or PCR test.

## References

Cheng, F., Luo, Y. and Naidu, R. 2022. Raman imaging combined with an improved PCA/algebra-based algorithm to capture microplastics and nanoplastics. Analyst.
